# The Landscape of Ferroptosis-Related Gene Signatures as Molecular Stratification in Triple-Negative Breast Cancer

**DOI:** 10.3390/diagnostics16030379

**Published:** 2026-01-23

**Authors:** Marko Buta, Nikola Jeftic, Irina Besu, Jovan Raketic, Ivan Markovic, Ana Djuric, Nina Petrovic, Tatjana Srdic-Rajic

**Affiliations:** 1 Surgical Oncology Clinic, Institute for Oncology and Radiology of Serbia, Pasterova 14, 11000 Belgrade, Serbia; 2 School of Medicine, University of Belgrade, Dr Subotica 8, 11000 Belgrade, Serbia; 3 Department of Experimental Oncology, Institute for Oncology and Radiology of Serbia, Pasterova 14, 11000 Belgrade, Serbia

**Keywords:** ferroptosis, triple-negative breast cancer, molecular stratification, multi-omics analysis, Ferroptosis Index, single-cell RNA sequencing

## Abstract

**Background:** Triple-negative breast cancer (TNBC) represents the most aggressive breast cancer subtype, characterized by high genomic instability, metabolic stress, and limited therapeutic options. Ferroptosis, an iron-dependent form of regulated cell death, has emerged as a promising vulnerability in TNBC, yet its subtype-specific regulatory landscape remains insufficiently defined. **Methods:** Using transcriptomic (METABRIC, TCGA, GEO) and proteomic (CPTAC) datasets, ferroptosis-related genes were profiled across PAM50 breast cancer subtypes. Differential expression, univariate Cox regression, LASSO modeling, survival analyses, GSEA, and dimensionality reduction (PCA, t-SNE) were applied. A Ferroptosis Index (FI) was calculated using β-coefficients from the Cox/LASSO regression model. Single-cell RNA-seq data was used to map ferroptosis-associated signature across tumor and microenvironmental compartments. **Results:** Basal-like tumors exhibited the strongest ferroptosis-associated transcriptional shift, characterized by upregulation of ACSL4 and EZH2 and downregulation of AR, GPX4, and CIRBP. Sixteen ferroptosis-related genes were associated with overall survival, forming a ferroptosis-associated signature. The FI was significantly higher in Basal-like tumors, indicating elevated ferroptosis-associated transcriptional state. GSEA revealed enrichment of cell cycle, mitotic, cytoskeletal, and metabolic stress pathways. Single-cell analysis demonstrated expression of ferroptosis markers across cancer epithelial, stromal, and myeloid populations. **Conclusions:** Basal-like tumors harbor a distinct ferroptosis-associated transcriptional state linked to tumor aggressiveness and poor prognosis. These findings provide a biologically grounded framework for ferroptosis-related stratification and support future functional and translational studies targeting ferroptosis vulnerabilities in aggressive breast cancer.

## 1. Introduction

According to the International Agency for Research on Cancer (IARC), breast cancer (BC) is the second-most frequent cancer worldwide, with 2.3 million new cases in 2022 [1]. It remains the leading cause of cancer mortality in women, with 666,103 deaths in 2022. Countries with a low Human Development Index (HDI) face a 17% higher mortality rate [2]. Despite advances in screening programs, early diagnosis, and access to treatment, the global burden of breast cancer continues to rise. A study by J. Kim et al. projected that by 2050, new BC cases and deaths related to BC will have increased by 38% and 68%, respectively, impacting low-HDI countries, implying that continued progression in BC management in early diagnosis and access to treatment is needed in countries with low and medium HDI [3].

The BC is a highly heterogeneous disease and classified into distinct molecular subtypes, including Luminal A, Luminal B, HER2-enriched, and Basal-like, which are clinically relevant. Among these, Basal-like BC, which largely corresponds to triple-negative breast cancer (TNBC), displays the most aggressive clinical course, characterized by early relapse, poor prognosis, and lack of targeted therapeutic options due to the absence of hormone receptors and HER2 amplification. Therefore, identifying molecular markers and prognostic models that can stratify patients and inform therapeutic strategies is particularly important in this subtype.

Although significant progress has been made in understanding and treating TNBC, the combination of late diagnosis, absence of effective endocrine and targeted therapies, and the inherently aggressive nature of the disease continues to result in poor clinical outcomes [4]. Currently, radiotherapy, immunotherapy, and targeted therapy are substantially reshaping the therapeutic landscape of TNBC [5]. Identifying more precise and effective molecular targets represents a promising avenue toward personalized treatment strategies for TNBC.

However, it has become increasingly evident that tumor cells could develop chemoresistance to apoptosis-dependent therapies and substantially increase the risk of treatment failure and post-treatment relapse. Therefore, targeting non-apoptotic cell death pathways, such as ferroptosis, in both basic cancer research and clinical studies offers a promising strategy to overcome resistance and develop more effective, durable cancer treatments that address critical unmet clinical needs.

In recent years, ferroptosis has emerged as a critical process in cancer biology. Unlike apoptosis or necroptosis, ferroptosis is closely linked to tumor metabolism, oxidative stress, and redox imbalance, processes often dysregulated in breast cancer [6]. Ferroptosis involves iron accumulation within the cell and abnormal lipid peroxidation, especially membrane lipids. A decrease in the cell’s antioxidant capacity and the accumulation of reactive oxygen species (ROS) ultimately lead to programmed oxidative cell death [7].

Accumulating evidence suggests that ferroptosis plays a dual role: it may act as a tumor suppressor mechanism by eliminating malignant cells, but can also contribute to tumor progression and therapy resistance. Consequently, ferroptosis-related genes (FRGs) have gained attention as potential biomarkers for cancer prognosis and therapeutic response. Different preclinical studies are confirming the efficacy of ferroptosis-based cancer therapies, but translating these findings into clinical trials remains limited [8].

The role of ferroptosis in BC subtypes, particularly TNBC, remains poorly characterized. Previous studies have highlighted individual genes involved in ferroptosis regulation, but systematic comparisons of ferroptosis-related transcriptional programs across BC subtypes are lacking. Moreover, robust ferroptosis-based prognostic signatures that can reliably stratify Basal-like patients into clinically meaningful risk groups have not been fully developed.

Triple-negative breast cancer (TNBC) exhibits a distinct metabolic and redox profile that makes it particularly susceptible to ferroptosis [9]. In particular, these tumors often display elevated intracellular iron levels and dysregulated iron metabolism [10,11], which enhance Fenton reactions and lipid peroxidation. They are also rich in polyunsaturated phospholipids (PUFA-PLs) [10,11], providing substrates for lipid peroxidation, and possess a distinct metabolic balance of iron and glutathione (GSH), which may render them more susceptible to ferroptosis [12]. Indeed, TNBC appears to be more sensitive to ferroptotic cell death compared with other breast cancer subtypes [13]. Moreover, ferroptosis inducers, such as GPX4 inhibitors, have emerged as potential therapeutic agents for TNBC [14]. Many studies have demonstrated that inducing ferroptosis by directly depleting the glutathione (GSH) depot, by blocking the Xc-system, which ultimately decreases intracellular GSH, or by inhibiting glutathione peroxidase 4 (GPX4), has promising potential for overcoming drug resistance in BC [9]. The main mechanism of ferroptosis differs from that of other types of cell death, which are usually targeted by most anticancer drugs. Given the lack of targeted therapies for this aggressive subtype, ferroptosis has emerged as a promising therapeutic vulnerability in TNBC.

However, the molecular heterogeneity of TNBC and the context-dependent regulation of ferroptosis highlight the need for systematic, data-driven approaches to identify ferroptosis-associated vulnerabilities with prognostic and therapeutic relevance. In this context, bioinformatics-based analyses have become increasingly important for ferroptosis research by enabling the systematic identification of ferroptosis-related gene signatures, resistance markers, and pathway-level vulnerabilities across cancer types [15,16,17,18,19]. Integrative transcriptomic approaches are widely used to link ferroptosis-associated genes, such as SLC7A11, GPX4, ACSL4, and lipid metabolism regulators, to patient prognosis, therapeutic response, and treatment resistance, thereby supporting mechanistic insight and rational target prioritization. Importantly, bioinformatics analyses are increasingly used to connect ferroptosis-related vulnerabilities with mechanism-driven therapeutic strategies. Recent studies show that targeting key ferroptosis pathways, such as iron metabolism, lipid peroxidation, and antioxidant defense, can be effectively combined with advanced drug delivery systems or multimodal treatment approaches in order to enhance treatment efficacy and support the development of combination therapies, including immunotherapy-based approaches [20,21].

This study aimed to characterize ferroptosis-related molecular features across breast cancer subtypes, with a particular focus on the Basal-like subtype. We further sought to identify ferroptosis-associated gene signatures that distinguish Basal-like tumors from other molecular subtypes and to develop a data-driven ferroptosis-based stratification framework associated with survival, with the goal of highlighting biologically relevant vulnerabilities and potential therapeutic targets.

## 2. Materials and Methods

### 2.1. Patient Cohorts and Clinical Data

Clinical, pathological, and survival data for breast cancer patients were obtained from the Molecular Taxonomy of Breast Cancer International Consortium (METABRIC cohort through the cBioPortal for Cancer Genomics. Patients were classified into molecular subtypes according to the PAM50 classification system, including Claudin-low, Luminal A, Luminal B, Normal-like, HER2-enriched, Basal-like, and not classified (NC). Clinical variables analyzed in this study included molecular subtype classification according to the PAM50 system, gene expression data, and overall survival. A summary of METABRIC patient clinical characteristics stratified by PAM50 molecular subtypes is provided in Supplementary Table S1. Only samples with available molecular subtype annotation and complete clinical and survival information were included in downstream analyses. Other clinicopathological variables available in the source datasets were not analyzed.

The METABRIC cohort comprised 209 Basal-like cases [22]. TCGA-BRCA and GEO cohorts were used for external evaluation and included TCGA *n* = 1081 and GEO *n* = 104 samples, following dataset-specific filtering criteria for available clinical and survival information. The CPTAC proteomics cohort included a total of 124 samples (Basal-like *n* = 29, Luminal A *n* = 57, Luminal B *n* = 17, HER2-enriched *n* = 16, Normal-like *n* = 5). Single-cell RNA-seq analysis (GSE176078) comprised TNBC samples from 10 samples.

### 2.2. METABRIC, TCGA, and GEO Datasets

#### Dataset Collection and Filtering

The cBioPortalData package was used to download mRNA data for METABRIC samples using the cBioPortal API. Corresponding clinical data were retrieved and filtered. Samples classified as Basal, Luminal A, Luminal B, HER2-enriched, and Normal-like according to the PAM50 classifier were included for downstream analyses.

The PAM50 Normal-like subtype was analyzed as an intrinsic breast cancer subtype characterized by relatively low proliferative activity and was not used as a surrogate for non-malignant breast tissue, but rather as a comparative reference within tumor heterogeneity.

To ensure consistency and comparability across heterogeneous transcriptomic cohorts, expression data were filtered to include only samples with complete molecular subtype annotation, gene expression data, and available survival information.

While the overall filtering strategy was consistent across datasets, cohort-specific criteria were applied depending on data availability (molecular subtype information in METABRIC, survival annotation in GEO, and sample size constraints in TCGA). This approach minimized technical noise and cohort heterogeneity while retaining biologically informative variation.

METABRIC data sources (https://www.cbioportal.org/study/summary?id=brca_metabric) were accessed on 23 January 2025; The Cancer Genome Atlas (TCGA, https://www.cancer.gov) data sources were accessed on 27 January 2025; Gene Expression Omnibus (GEO, https://www.ncbi.nlm.nih.gov/geo/) data sources were accessed on 28 January 2025; GSE176078 data sources were accessed on 4 February 2025. All analyses were performed in R (version 4.4.1) using Bioconductor (version 3.20).

### 2.3. DEG Analysis

For differential gene expression analysis (DGE) between Basal and Normal-like BCs, the limma package was used. Design matrices for linear modeling were created, and eBayes correction was implemented for statistical analysis. Only statistically significantly differentially expressed genes (*p*-value < 0.05 and log2 expression change greater than 1) were filtered (Supplementary Tables S2–S5).

### 2.4. Analysis and Visualization of Ferroptosis Markers

Ferroptosis markers were selected from previous analysis. These 509 selected ferroptosis markers were obtained from the literature and online databases [23,24]. Then, ferroptosis markers differentially expressed between Basal and Normal-like BCs were selected. A heatmap was created to visualize DEGs of ferroptosis genes using the heatmap package. A CSV tabular file containing only significant ferroptosis-differential genes was also created.

### 2.5. Univariate Cox Regression Model for the Basal Cohort

The association between gene expression and survival endpoints in the Basal cohort was assessed using univariate Cox regression. Hazard ratio (HR) and *p*-values were generated for each gene. Results were filtered for genes with *p*-values less than 0.05 (Supplementary Table S6). Only ferroptosis markers (16 of them) were selected.

### 2.6. Generation of a Venn Diagram

The Venn Diagram package was used to generate a Venn diagram showing the intersection between differentially expressed genes (DEGs) and prognostic genes associated with ferroptosis.

### 2.7. LASSO Regularization for Cox Regression

The glmnet library for LASSO regression was used to identify relevant genes. As an input for LASSO, the union of genes from the Venn diagram was used. A visualization of the LASSO coefficients was created, and the minimum lambda value was selected for the model. β-coefficients derived from the LASSO Cox model were used exclusively for feature selection and relative weighting within the risk score, and were not interpreted as independent prognostic effect sizes.

### 2.8. Kaplan–Meier Analysis

Kaplan–Meier survival curves were generated using the survival package based on the calculated risk score. Differences between risk groups were assessed using the log-rank test. The TCGA cohort and the GEO database set (GSE42568) were used for external evaluation of this model.

### 2.9. ROC Curve

Time-dependent Receiver Operating Characteristic (ROC) curves were calculated at 12, 36, and 60 months to assess the discriminatory performance of the risk score. ROC analyses were interpreted as measures of biological risk stratification rather than clinical prediction.

### 2.10. PCA and t-SNE Analysis

A PCA (Principal Component Analysis) was conducted based on the expression of selected prognostic genes, and visualized by representing high- and low-risk groups, with an emphasis on the difference between them; a t-SNE plot was also generated.

### 2.11. GSEA Between High and Low Risk Groups

Using previously obtained data, gene expression, and information on risk groups, we performed differential gene expression analysis followed by gene set enrichment analysis between high- and low-risk groups. For the linear fitting model, lmFit and eBayes functions were used to fit a linear model with the appropriate design, based on the risk group. For the DGE analysis, a list of genes was created showing differential expression between high- and low-risk groups based on a model coefficient. Genes were mapped from symbols to ENTREZ IDs for gene ontology (GO) and Kyoto Encyclopedia of Genes and Genomes (KEGG) pathway analysis using gseGO and gseKEGG functions. Tabular CSV files and dot plot figures were generated for the most significant categories.

### 2.12. FI Calculation

The Ferroptosis Index (FI) was calculated as a weighted sum of ferroptosis-related gene expression values using the corresponding β-coefficients derived from the univariate Cox/LASSO regression model:

FI = Σ (βᵢ × expressionᵢ)

β-coefficients were derived from the METABRIC cohort and reflect the direction and relative contribution of individual genes to survival-associated ferroptosis-related transcriptional programs. The FI was calculated solely within the METABRIC cohort and was neither recalibrated nor externally validated as a predictive score. Genes included in the FI were selected to represent both ferroptosis drivers and suppressors across key regulatory pathways, integrating survival-associated differentially expressed genes with well-established ferroptosis markers to ensure biological interpretability rather than prognostic optimization. Higher FI values indicate a ferroptosis-prone transcriptional state, whereas lower values correspond to a ferroptosis-resistant phenotype.

### 2.13. Gene and Protein Expression Analysis

mRNA expression and clinical data from the METABRIC cohort were retrieved and analyzed using the cBioPortalDataR package via the cBioPortal version 6.0 API for Cancer Genomics. Protein expression data from CPTAC were obtained through the cBioPortalDataR package via the cBioPortal API. Gene expression comparisons, subtype analyses, and Kaplan–Meier survival curves were generated directly within the cBioPortal using their built-in statistical modules. Only genes with complete expression and survival information were included in downstream interpretation. All databases and online platforms were accessed on 23 September 2025. Multiple testing correction was not applied to gene-level survival analyses, as these analyses were hypothesis-driven and restricted to a predefined set of ferroptosis-related genes. Results should therefore be interpreted in a biological and exploratory context. All analyses were performed in R (version 4.5.1) using Bioconductor (version 3.21).

### 2.14. Single-Cell Sequencing Analysis

For single-cell sequencing analysis (scRNA-seq), the publicly available GSE176078 dataset was used, which contains information on triple-negative breast cancer samples. The purpose of this analysis was to find out the cell types with the most abundant ferroptosis marker gene transcripts from our prognostic markers (16 ferroptosis-related genes). The Seurat package was used for data processing and cluster visualization, as well as for the identification of ferroptosis markers. After the Seurat object was created, metadata on samples and cell type were added, and TNBC samples were selected. Subsequently, data normalization was performed, variable genes were found, the data were scaled, and PCA was applied.

In the following steps, dimensionality reduction was performed using uniform manifold approximation and projection UMAP and t-SNE techniques, thus obtaining visually better clusters. Clusters have been pre-identified and ANNOTATED by the dataset creator, and there were two annotations—major and minor, depending on the depth of the cell subtype investigation. Further analyses were performed with both major and minor. Furthermore, ferroptosis markers for different cell types (major and minor types) in TNBC samples were identified (gene count in each cell subtype). The point of this approach was to determine whether the markers identified by the prognostic model are present in the scRNA-seq model and whether they are characteristic of specific cell types. These markers were assessed across different cell types and visualized using various graphical representations (UMAP, VlnPlot, DotPlot, FeaturePlot). Two ferroptosis-related genes H19 and CGAS (out of 16 from DEG analysis) were not present (annotated) in this dataset, and the analysis was performed on fourteen available genes.

## 3. Results

### 3.1. Ferroptosis-Related Gene Expression Across Breast Cancer Subtypes

**Figure 1:**
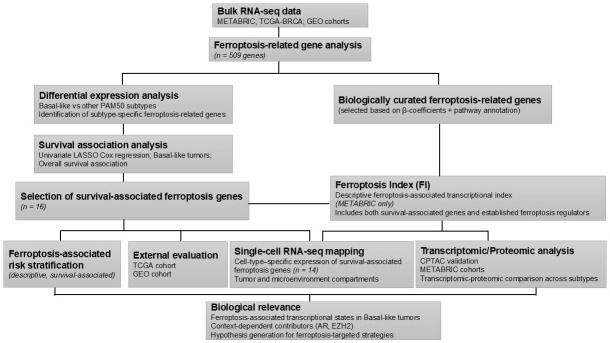
Schematic overview of the analytical workflow. Overview of the analytical workflow integrating bulk transcriptomic, proteomic, and single-cell RNA-seq analyses to characterize ferroptosis-associated transcriptional states in Basal-like tumors.

To evaluate the expression patterns, survival-associated stratification, and biological pathways related to ferroptosis across breast cancer subtypes, comprehensive analyses were conducted. The ferroptosis gene panel analysis revealed distinct expression patterns across breast cancer subtypes, underscoring the heterogeneity of ferroptosis regulation within the disease. To further explore these molecular differences, integrative transcriptomic and proteomic analyses were conducted across the METABRIC, TCGA, GEO, and CPTAC cohorts. An overview of the analytical workflow integrating bulk transcriptomic, proteomic, and single-cell analyses, from ferroptosis gene curation to survival modeling and biological interpretation, is summarized in Figure 1.

**Figure 2:**
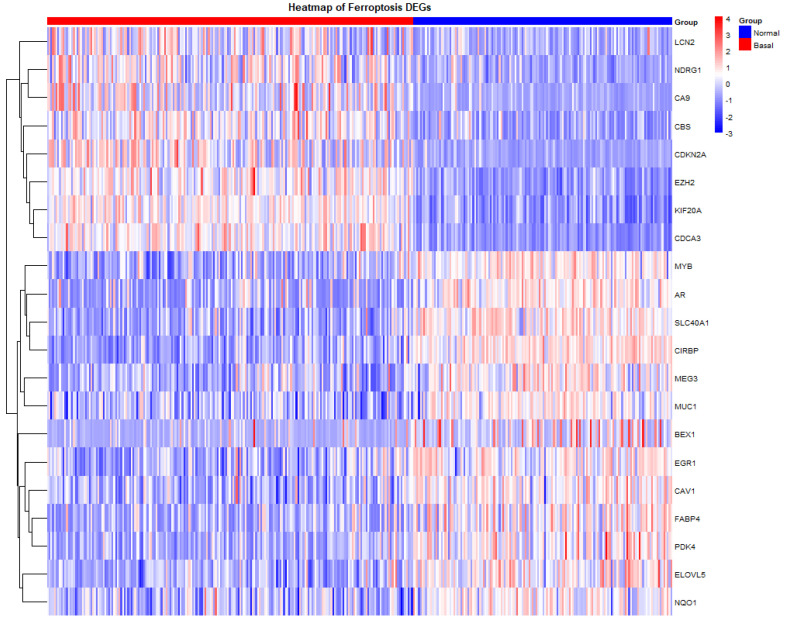
Expression heatmap of ferroptosis-related genes in Basal-like and Normal-like tumors. Heatmap illustrating the expression patterns of ferroptosis-related genes across Basal-like (red) and Normal-like (blue) samples, showing clear group-level segregation and a distinct ferroptosis-associated signature enriched in basal-like tumors.

The heatmap visualization demonstrated apparent differences in ferroptosis gene expression between Normal-like and Basal breast cancer groups (Figure 2). Specifically, LCN2, NDGR1, CA9, CBS, CDKN2A, EZH2, KIF20A, and CDCA3 were significantly overrepresented in the Basal group and underrepresented in the Normal-like group. Opposingly, MYB, AR, SLC40A1, CIRBP, MEG3, MUC1, BEX1, EGFR1, CAV1, FABP4, PDK4, ELOVL5, and NQO1 were prominently under-represented in the Basal group, but over-represented in most samples analyzed in the Normal-like group. Gene clustering analysis revealed that specific genes, including NDRG1 and CA9, as well as KIF20A and CDCA3, tend to co-overexpress in the Basal group. To extend the analysis beyond the Basal-like group, the expression of ferroptosis-related genes was compared among the other molecular subtypes of breast cancer, including Luminal A, Luminal B, and HER2-enriched, using METABRIC transcriptomic data (Figure S1).

When comparing Luminal A versus Basal BC, clustering of samples demonstrates not so clear separation between the groups, with basal tumors displaying slight upregulation of several ferroptosis regulators, including TF, PLIN2, IDO1, LCN2, MT1G, EGFR, LPIN1 and NDRG1 (Figure S1A).

Similarly, in the comparison of Luminal B versus Basal BC, no consistent divergence is observed (Figure S1B). Genes such as MT1G, NDRG1, EGFR, CA9, LCN2, NT5DC2, CBS, TF, LPIN1 and CDKN2A show slightly enriched expression in Basal cancers, whereas Luminal B tumors exhibit comparatively lower or heterogeneous expression across many ferroptosis-related markers (Figure S1B).

A clear separation between the Basal and HER2 groups is evident, indicating distinct ferroptosis transcriptional landscapes (Figure S1C). Basal-like tumors exhibit higher expression of LCN2, CDKN2A, FZD7, and TF (Figure S1C). Conversely, HER2-enriched tumors exhibit increased expression of SLC40A1, NQO1, AR, and MUC1.

**Figure 3:**
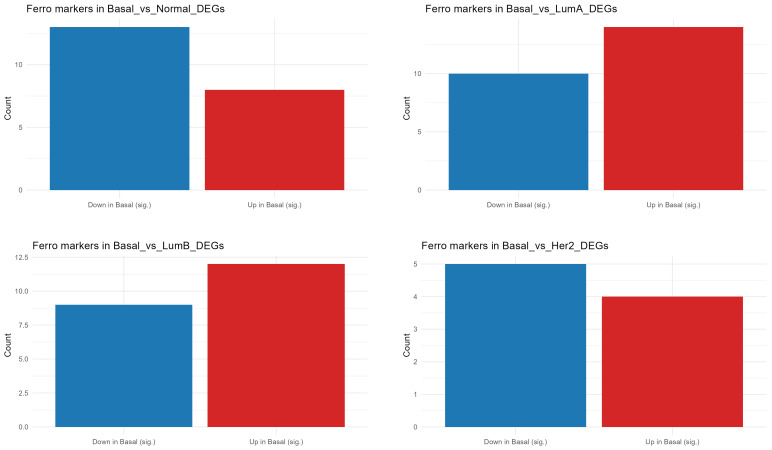
Bar plots showing the number of significantly upregulated (red) and downregulated (blue) ferroptosis-related genes in Basal-like tumors compared with other PAM50 breast cancer subtypes (Luminal A, Luminal B, HER2-enriched, and Normal-like). Basal-like tumors exhibit the highest proportion of dysregulated ferroptosis-related genes, suggesting a more substantial ferroptosis-associated transcriptional shift than other subtypes.

The bar plots presented in Figure 3 illustrate the distribution of significantly upregulated (red) and downregulated (blue) ferroptosis-related genes in Basal-like tumors compared with other breast cancer subtypes (Luminal A, Luminal B, HER2-enriched, and Normal-like). Overall, the number of ferroptosis genes upregulated in basal tumors is higher compared to the Luminal A and Luminal B subtypes, indicating increased ferroptosis susceptibility at the transcriptional level in these highly proliferative and oxidative phenotypes (Figure 3, Table S3 and Table S4).

In contrast, fewer ferroptosis markers are upregulated in HER2-enriched versus Basal and more are downregulated compared to Normal-like tumors, suggesting that Basal tumors exhibit a distinct ferroptotic profile characterized by selective activation of oxidative and iron-related pathways (Figure 3 and Table S5).

#### 3.1.1. Identification of Ferroptosis-Related Markers Associated with Survival in Basal-Like Tumors

**Figure 4:**
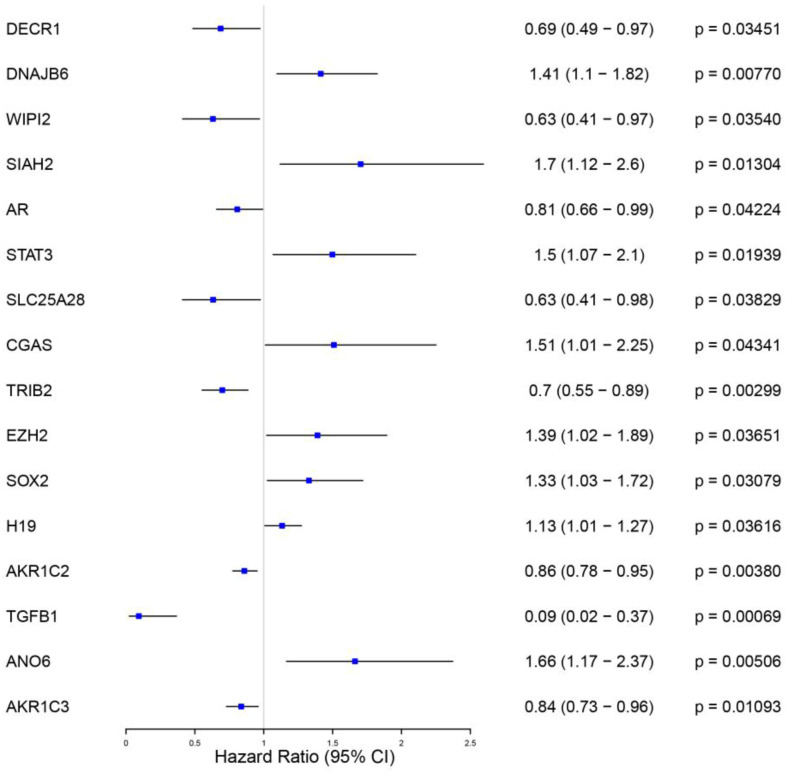
Univariate Cox proportional hazards analysis of ferroptosis-related genes. Forest plot illustrating hazard ratios (HRs), 95% confidence intervals, and *p*-values for ferroptosis-related genes associated with overall survival in the METABRIC Basal-like cohort. Genes with HR > 1 indicate increased risk, while those with HR < 1 indicate protective associations.

**Figure 5:**
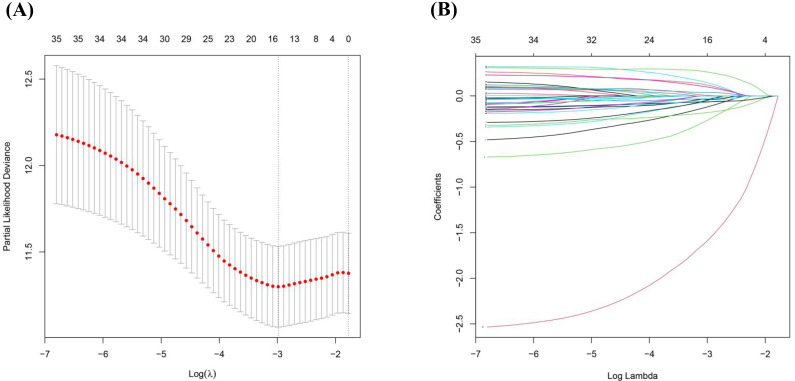
LASSO Cox regression for selection of prognostic ferroptosis-related genes. (**A**) represents partial likelihood deviance in the function of the logarithm of λ parameter (logλ). The left dotted line represents the minimum mean value of the partial likelihood deviance (λ) in this model. The right dotted line is the λ value of the simplest model from the variance range. (**B**) Coefficients of LASSO model.

After identifying differentially expressed genes between Basal and other tumor subtypes in the METABRIC dataset, the analysis focused on identifying ferroptosis-related genes associated with overall survival to enable biological risk stratification within Basal-like tumors. These genes were further evaluated using univariate Cox regression and LASSO regularization to construct a ferroptosis-associated stratification model (Figure 4 and Figure 5).

A forest plot was used to present the association between ferroptosis-related gene expression and survival in the Basal subgroup after the univariate Cox proportional hazards analysis. Hazard ratio (HR) and *p*-values were calculated for each ferroptosis-related gene. Effect sizes are reported as HRs with corresponding 95% confidence intervals derived from univariate Cox proportional hazards models. The HR value reflects the association between gene expression, and survival. This analysis showed that eight genes (DECR1, WIPI2, AR, SLC25A28, TRIB2, AKR1C2, TGFB1, and AKR1C3) were negatively associated with survival time and favorable outcome, and better prognosis, while the other eight ferroptosis-related genes with HR above one (DNAJB6, SIAH2, STAT3, CGAS, EZH2, SOX2, H19, ANO6) were associated with death event and poorer prognosis (Figure 4). According to the results presented in Figure 4, the TGFB1 gene may have the most significant protective effect on survival endpoints.

**Figure 6:**
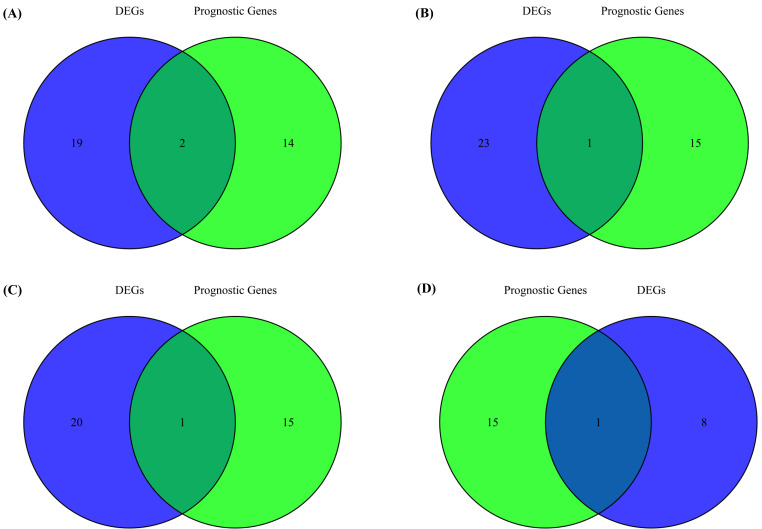
Overlap between prognostic ferroptosis-related genes associated with survival and differentially expressed ferroptosis-related genes across breast cancer subtypes. Venn diagrams showing the intersection between prognostic genes and differentially expressed ferroptosis-related genes in Basal-like versus (**A**) Normal-like, (**B**) Luminal A, (**C**) Luminal B, and (**D**) HER2-enriched subtypes. Basal-like tumors exhibit the most significant overlap, highlighting stronger ferroptosis-linked prognostic dependencies in TNBC.

Furthermore, to identify overlapping genes between ferroptosis-related genes associated with survival and those differentially expressed across molecular subtypes, a Venn diagram analysis was conducted. The results showed that AR was the only gene to overlap consistently across all subtype comparisons, whereas EZH2 additionally overlapped in the Basal vs. Normal-like comparison (Figure 6). This finding highlights AR as the most consistently identified ferroptosis-associated gene within the Basal subtype.

#### 3.1.2. Construction and Evaluation of Ferroptosis-Associated Transcriptional Stratification Framework

**Figure 7:**
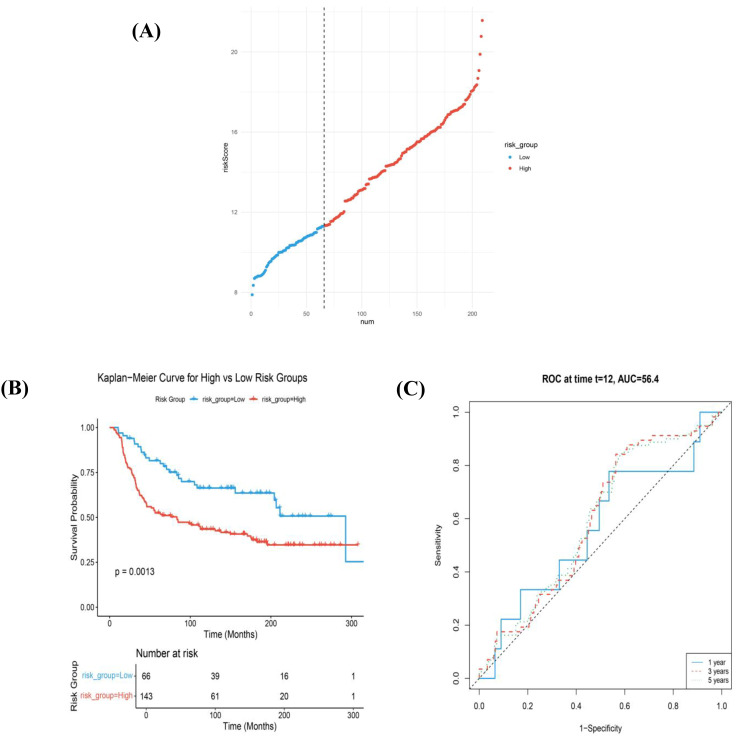
Construction and evaluation of the ferroptosis-associated transcriptional stratification framework for Basal-like tumors. From top to bottom were the risk score distribution (**A**) and the overall survival status of patients across low- and high-risk groups (**B**,**C**). Breast cancer patients were divided into low- and high-risk groups based on the METABRIC cohort cut-off values. (**B**) Kaplan–Meier survival curves comparing low- vs. high-risk signatures in the METABRIC cohort and (**C**) time-dependent ROC curves demonstrating predictive performance of the risk score model at different time points.

The ferroptosis-based risk model was developed in the Basal-like subtype due to its pronounced ferroptosis-associated transcriptional profile and association with clinical outcome. A risk score was calculated by multiplying each gene’s expression by its corresponding coefficient. It was further used to create binary risk groups (high-risk and low-risk groups) for METABRIC basal cohort (Figure 7A), TCGA, and GEO external evaluation cohort (Figure S2A,B). Based on the optimized risk score threshold (the cut point was calculated with the survival cut point function), patients were divided into two groups—high and low (Figure 7). The same coefficients and cut point were applied unchanged to the TCGA and GEO cohorts for external evaluation (Figure S2A,B).

Kaplan–Meier survival plots were calculated based on risk score, between the high- and low-risk groups. A significantly higher survival probability was observed in the low-risk group (*p* = 0.0013) (Figure 7B). To estimate the predictive accuracy of the calculated risk score at 12, 36, and 60 months, ROC analyses were performed across the three cohorts. In the METABRIC cohort, the area under the curve (AUC) was 56%, indicating modest discriminatory ability and supporting interpretation of the model as a biologically informative stratification framework rather than an independent clinical prognostic tool (Figure 7C).

In the TCGA evaluation cohort, low-risk patients also showed a significantly improved overall survival (*p* = 0.043) (Figure S3A). In contrast, no statistically significant survival difference was observed in the GEO cohort (*p* = 0.11), probably due to limited sample size and cohort heterogeneity (Figure S3B). Due to the small number of investigated samples, the AUC could not be estimated (Figure S3B). Collectively, these findings indicate that the proposed model provides supportive, rather than definitive, evidence of ferroptosis-associated risk stratification in Basal-like breast cancer.

#### 3.1.3. Dimensionality Reduction Analysis of Risk Groups

Principal component analysis (PCA) and t-distributed stochastic neighbor embedding (t-SNE) were performed to assess the separation between high- and low-risk groups based on the ferroptosis-related gene signature. The dimensionality of the gene expression interplay was investigated using principal component analysis (PCA). PCA uses global image and topographic information, focusing on the whole-cohort variance and preserving point-to-point distances, while t-SNE, as a non-linear dimensionality reduction method, also projects high-dimensional data into a lower-dimensional space (2D or 3D). Still, it converts high-dimensional pairwise similarities into probabilities, minimizes differences in these probabilities across high- and low-dimensional spaces to push similar points closer together while pushing dissimilar points apart, uses gradient descent to optimize the embedding, and maintains the locality of relations. Both approaches revealed no distinct clustering between the two groups, indicating only subtle transcriptomic heterogeneity (Figure S4).

### 3.2. Gene Set Enrichment Analysis of Risk Groups and Molecular Subtypes

To better understand the biological context of the identified genes, gene set enrichment analysis (GSEA) was applied to both risk groups and molecular subtypes. This revealed that ferroptosis-associated genes were predominantly linked to cell cycle progression, mitotic activity, and adaptation to metabolic stress, suggesting a mechanistic role in maintaining the aggressive Basal-like phenotype. Gene Set Enrichment Analysis (GSEA) was performed for GO Biological Process and KEGG pathways using the gseGO and gseKEGG functions. In all plots, color intensity represents the adjusted *p*-value, while node diameter corresponds to the gene set size.

**Figure 8:**
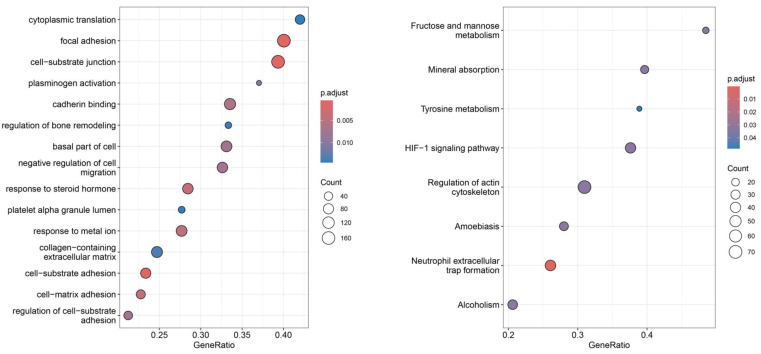
Functional enrichment analysis of consensus ferroptosis-related genes. GO biological process enrichment identifies processes involving focal adhesion, cell–substrate junction, and cell–substrate adhesion, indicating remodeling of cell–matrix interactions (**left**). KEGG pathway enrichment indicates neutrophil extracellular trap formation, HIF-1 signaling, fructose and mannose metabolism, reflecting activation of hypoxia-driven and metabolic stress responses (**right**).

In the analysis of high- and low-risk groups within Basal tumors, the most significantly enriched GO terms were focal adhesion, cell–substrate junction, and cell–substrate adhesion, indicating remodeling of cell–matrix interactions. According to KEGG pathway enrichment, the top-ranked pathways included neutrophil extracellular trap formation, HIF-1 signaling, fructose and mannose metabolism, and mineral absorption, reflecting activation of hypoxia-driven and metabolic stress responses (Figure 8).

When focusing on ferroptosis-associated differentially expressed genes (DEGs) across molecular subtypes, GSEA revealed distinct enrichment patterns.

For Basal versus Normal-like tumors, ferroptosis-related DEGs were strongly enriched in cell cycle regulation and mitotic progression pathways (Figure S5A). The top GO terms include cell cycle process, chromosome segregation, and mitotic nuclear division and KEGG pathways include cell cycle, microRNAs in cancer, and cellular senescence, indicating links between ferroptosis, proliferation, and chromosomal dynamics and oncogenic signaling in Basal tumors (Figure S5A).

In Basal versus Luminal A, enrichment was again observed in cell cycle and checkpoint control pathways. Top GO terms (cell cycle process, chromosome segregation, mitotic division) (Figure S5B) and KEGG results (cell cycle, cellular senescence, p53 signaling pathway) indicate that ferroptosis-related genes in Basal tumors are functionally linked to enhanced cell division, chromosomal organization, and DNA metabolism (Figure S5B).

In Basal vs. Luminal B, enriched GO terms included supramolecular fiber organization, cytoskeleton organization, and epidermis development, suggesting roles in structural remodeling, differentiation, and metabolic reprogramming (Figure S5C). KEGG pathways such as Cushing syndrome, hepatocellular carcinoma, and microRNAs in cancer support the involvement of ferroptosis in stress response and oncogenic signaling (Figure S5C).

For Basal versus HER2, moderate enrichment was found in GO terms related to development and organelle organization, while KEGG identified cornified envelope formation and metabolic pathways (Figure S5D). These results imply that ferroptosis-related genes in Basal tumors contribute to developmental and metabolic adaptation.

### 3.3. Ferroptosis Index Analysis

To integrate multiple ferroptosis-related signals into a single variable, a Ferroptosis Index (FI) was calculated.

The FI represents a computational score that quantifies the overall ferroptosis-associated transcriptional state of each tumor sample based on the expression of ferroptosis-related genes. FI quantifies the balance between ferroptosis driver and suppressor gene expression in each tumor, and is used to estimate whether a tumor is more likely to exhibit a ferroptosis-prone or ferroptosis-resistant phenotype, providing insight into oxidative stress vulnerability and potential therapeutic sensitivity.

**Figure 9:**
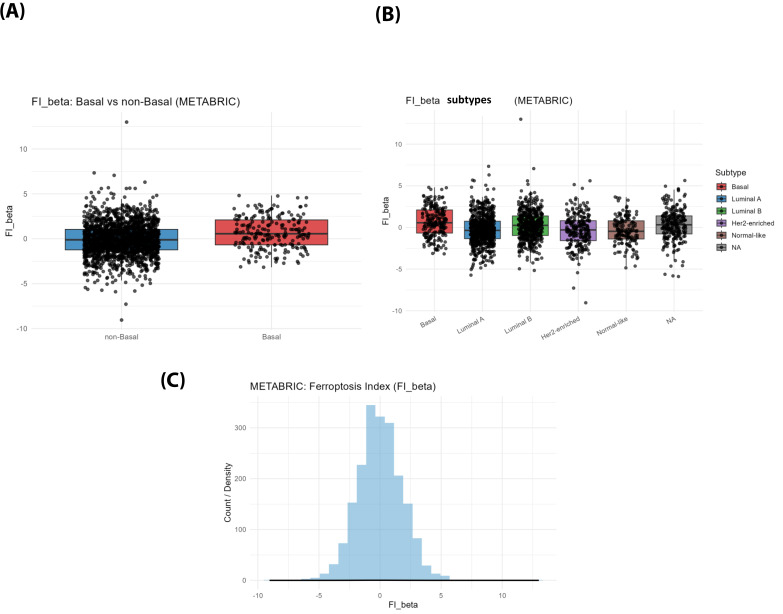
Ferroptosis Index (FI) across breast cancer subtypes. (**A**) An FI comparison between Basal and non-Basal tumors in the METABRIC cohort shows that Basal tumors have higher ferroptosis-associated transcriptional state. (**B**) FI distribution across PAM50 subtypes shows the highest FI in Basal tumors, followed by Luminal A, Luminal B, HER2-enriched, and Normal-like subtypes. (**C**) Overall, the FI distribution across the entire METABRIC cohort shows a roughly normal pattern, with a small number of extreme outliers. Together, these results indicate that Basal tumors exhibit a markedly elevated ferroptosis potential.

In the left panel, comparison between Basal and non-Basal breast cancers revealed a significantly higher FI in Basal tumors (median = 0.56, IQR = 2.78) compared to non-Basal (median = −0.11, IQR = 2.28), according to the Wilcoxon rank-sum test (W = 142797; *p* = 6.4 × 10^−8^). This demonstrates a markedly elevated ferroptosis-associated transcriptional state in the Basal subtype (Figure 9).

The middle panel shows FI across all intrinsic subtypes. The difference was highly significant (Kruskal–Wallis χ^2^ = 63.7, df = 4, *p* = 4.9 × 10^−13^). Post hoc pairwise tests confirmed that Basal tumors had significantly higher FI compared with Luminal A (*p* = 1.3 × 10^−9^), HER2-enriched (*p* = 7.3 × 10^−7^), and Luminal B (*p* = 6.8 × 10^−3^), and Normal-like (*p* = 8.2 × 10^−8^) subtypes (Figure 9).

The right panel illustrates the overall distribution of FI across the METABRIC cohort, following an approximately normal pattern centered near zero, with the majority of tumors falling between −3 and +3 (Figure 9). A small number of outliers (up to ±10) suggest the presence of rare subgroups exhibiting pronounced ferroptosis-related transcriptional regulation (Figure 9).

#### 3.3.1. mRNA Expression of Ferroptosis- and Signaling-Related Genes in Basal vs. Non-Basal Tumors

To gain deeper insight into the transcriptional basis of ferroptosis regulation, we analyzed the expression of ferroptosis- and signaling-related genes in Basal and non-Basal tumors from the METABRIC dataset. To analyze mRNA expression of ferroptosis- and signaling-related genes, we selected genes with distinct β-coefficients in the prognostic model, representing diverse functional categories and cellular processes associated with ferroptosis regulation and tumor progression. Analysis of mRNA expression (z-scores) in the METABRIC cohort revealed distinct ferroptosis- and stress-related transcriptional profiles between Basal and non-Basal breast tumors.

**Figure 10:**
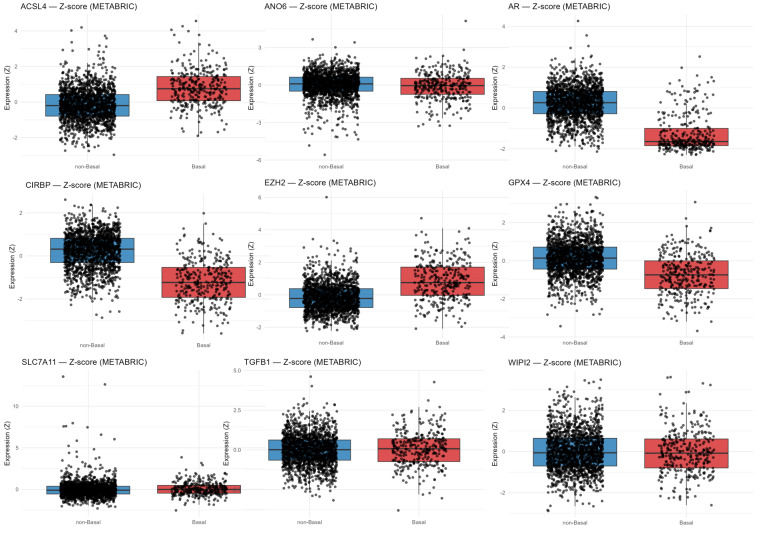
mRNA expression of ferroptosis- and signaling-related genes in Basal vs. non-Basal tumors (METABRIC). The displayed genes were selected for inclusion in the FI as both ferroptosis inducers or suppressors, and as components of signaling pathways associated with ferroptosis, including lipid peroxidation, iron handling, antioxidant defense, autophagy, and stress-response signaling. Gene selection was guided by model-derived β-coefficients and biological annotations, and complemented by established ferroptosis markers to enhance biological interpretability. Transcriptomic comparison of selected ferroptosis-related genes shows that ACSL4, and EZH2 are significantly upregulated in Basal tumors, whereas AR, GPX4, and CIRBP are strongly downregulated, reflecting a ferroptosis-prone, antioxidant-deficient transcriptional state characteristic of Basal-like tumors.

ACSL4 and EZH2 were significantly upregulated in Basal tumors, while AR, GPX4, and CIRBP were downregulated (Figure 10). In contrast, ANO6, SLC7A11, TGFB1, and WIPI2 showed no significant difference in expression between Basal and non-Basal tumors (Figure 10).

#### 3.3.2. Gene Expression Patterns of Ferroptosis-Related and Regulatory Markers Across Breast Cancer Subtypes

**Figure 11:**
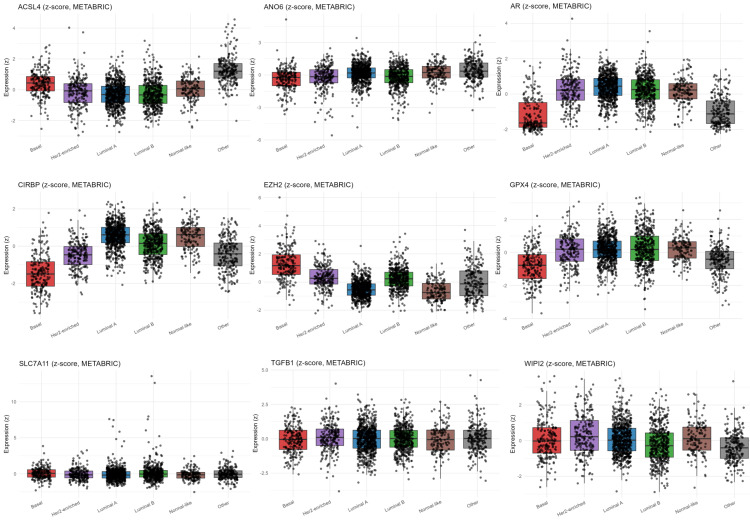
Expression patterns of ferroptosis-related markers across PAM50 breast cancer subtypes (METABRIC). ACSL4 and EZH2 are most highly expressed in Basal tumors, while AR, CIRBP and GPX4 exhibit the lowest expression, consistent with loss of hormonal signaling and reduced ferroptosis resistance in TNBC. ANO6, TGFB1, SLC7A11, and WIPI2 exhibit low, relatively uniform expression across subtypes.

To further delineate subtype-specific transcriptional differences, ferroptosis-related gene expression was next examined within individual PAM50 molecular subtypes in the METABRIC cohort. ACSL4 and EZH2 were markedly upregulated in Basal tumors, whereas other tumor subtypes showed lower variability and lower ACSL4 expression (Figure 11). EZH2 was lowest in Luminal A and Normal-like, while HER-2 enriched and Luminal B showed moderate expression (Figure 11). At the same time, AR, CIRBP, and GPX4 were strongly reduced in the Basal-like subtype, while Luminal A/B, HER-2 enriched, and Normal-like consistently show lower variability and higher expression of these genes (Figure 11). ANO6, TGFB1, SLC7A11, and WIPI2 remained low across all subtypes (Figure 11).

#### 3.3.3. Protein Abundance of Ferroptosis- and Signaling-Related Markers

To validate whether transcriptional changes translate to the protein level, CPTAC proteomic data were analyzed for ferroptosis-related markers.

**Figure 12:**
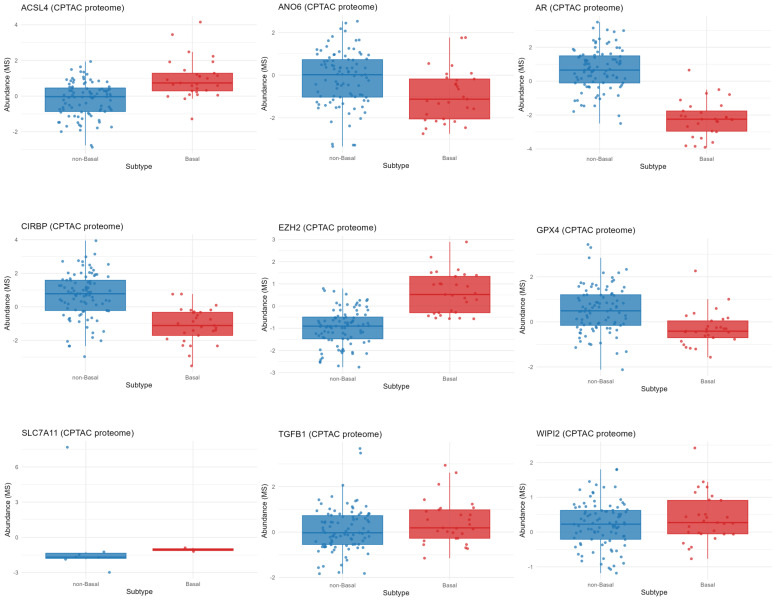
Protein abundance of ferroptosis- and signaling-related markers across Basal and non-Basal tumors (CPTAC). Proteomic profiling confirms transcriptomic trends, with elevated ACSL4 and EZH2 protein abundance in Basal tumors and markedly reduced levels of AR, GPX4, CIRBP, and ANO6. These findings support a ferroptosis-prone, epigenetically reprogrammed Basal phenotype with impaired antioxidant capacity.

ACSL4 and EZH2 showed higher protein abundance in Basal tumors (Figure 11). In contrast, ANO6, AR, GPX4, and CIRBP were markedly reduced (Figure 12). SLC7A11 expression was low, while WIPI2 showed a mild increase in Basal tumors (Figure 12).

#### 3.3.4. Proteomic Profiling of Ferroptosis-Related Markers Across PAM50 Subtypes

**Figure 13:**
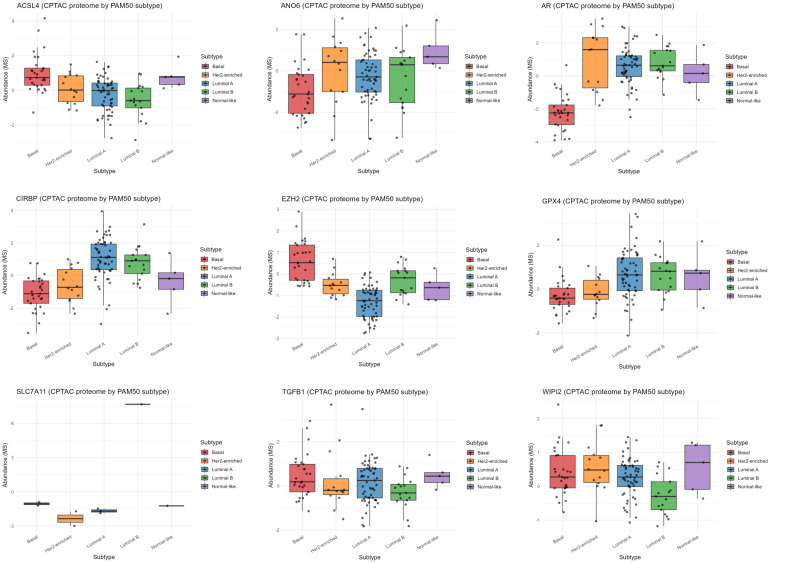
Proteomic profiling of ferroptosis-related markers across PAM50 subtypes (CPTAC). Basal tumors display the highest ACSL4 and EZH2 protein abundance and the lowest ANO6, AR, GPX4, and CIRBP levels, indicating a ferroptosis-prone state and loss of hormone-dependent redox control. Luminal tumors exhibit higher AR, GPX4, and CIRBP expression. WIPI2 is moderately elevated in Normal-like, Basal and HER2-enriched tumors, whereas SLC7A11 is highest in Luminal B.

Protein abundance of ferroptosis-related markers was next evaluated across PAM50 subtypes using the CPTAC dataset. ACSL4 and EZH2 were highest in Basal tumors (Figure 13). In contrast, AR and CIRBP showed the lowest expression in Basal and the highest in Luminal tumors (Figure 13). ANO6 peaked in Luminal A and Normal-like subtypes, while Basal tumors showed slightly lower levels (Figure 13). GPX4 was lowest in Basal and highest in Luminal tumors (Figure 13). WIPI2 was moderately elevated in Basal and HER2-enriched tumors, whereas SLC7A11 was generally low except in Luminal B, where it was markedly upregulated (Figure 13).

#### 3.3.5. Survival Analysis of Ferroptosis-Related Gene Expression Levels in the METABRIC Cohort

**Figure 14:**
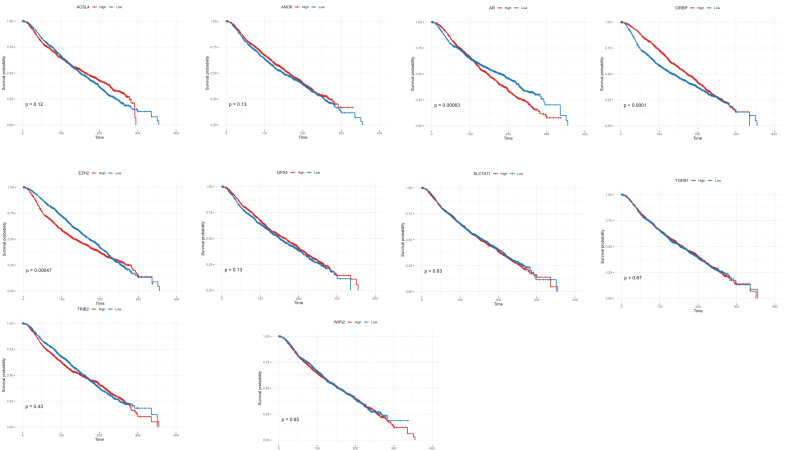
Gene-specific survival analyses of ferroptosis-related genes in Basal-like breast cancer. Kaplan–Meier curves showing overall survival differences associated with the expression of each ferroptosis-related gene in the METABRIC basal-like cohort.

To determine whether these molecular differences translate into clinical outcomes, we next evaluated the association between selected ferroptosis-related gene expression levels and overall survival in the METABRIC cohort. Survival analysis confirmed that EZH2 and AR overexpression and low CIRBP expression were significantly associated with poor overall survival (*p* < 0.001) (Figure 14). Univariate Cox proportional hazards analysis further supported these associations, with EZH2 (HR = 1.23, 95% CI: 1.10–1.38, *p* = 0.00047) and AR (HR = 1.23, 95% CI: 1.09–1.38, *p* = 0.00063) showing hazard ratios greater than 1, while CIRBP (HR = 0.78, 95% CI: 0.70–0.88, *p* < 0.001) exhibited a protective effect.

Other ferroptosis-related genes did not show statistically significant associations with overall survival, as their confidence intervals overlapped unity (Figure 14).

### 3.4. Single Cell Analysis

**Figure 15:**
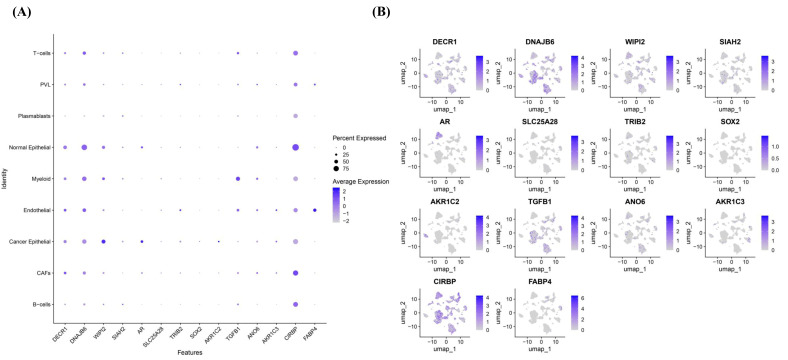

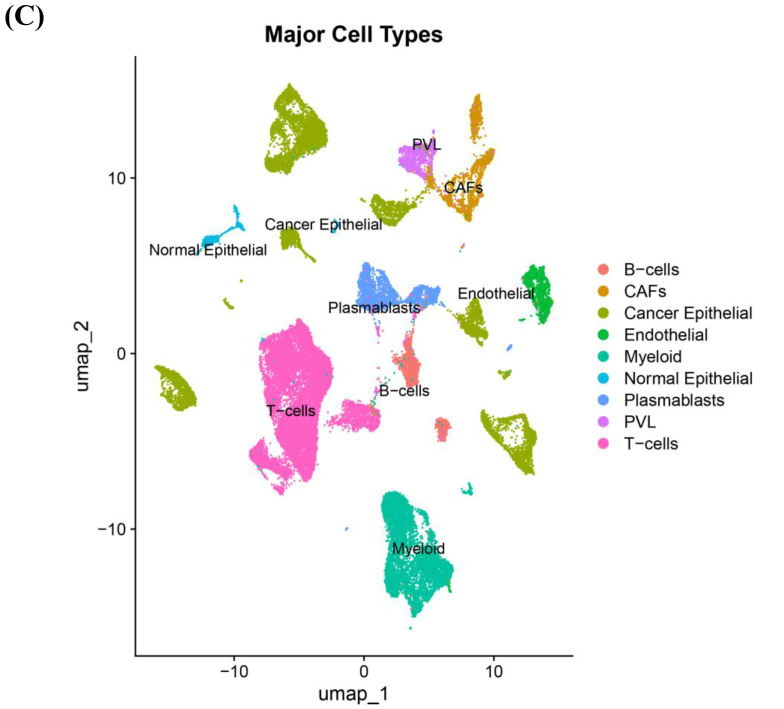
Expression of ferroptosis-related markers across major cell types in TNBC (scRNA-seq, GSE176078). (**A**) Dot plot showing expression level (color intensity) and percentage of expressing cells (dot size) across major cell types. (**B**) UMAP and (**C**) Seurat UMAP projections illustrating spatial clustering and distribution of ferroptosis-related transcripts. CIRBP, TGFB1, AR, and WIPI2 are predominantly expressed in cancer epithelial, CAF, endothelial, and myeloid cells, indicating multi-compartmental regulation of ferroptosis within the tumor microenvironment.

Single-cell RNA-seq analysis was performed to map ferroptosis-associated markers across tumor and microenvironmental compartments in TNBC using the GSE176078 dataset. Cell type annotation identified malignant epithelial cells, cancer-associated fibroblasts (CAFs), endothelial cells, T-cells, macrophages, and dendritic cells. Ferroptosis-related genes showed distinct and cell-type-specific expression patterns, indicating that ferroptosis-associated transcriptional signals are detected not only in tumor cells but also across stromal and immune populations (Figure 15A–C).

Expression analysis across major cell types showed that CIRBP, TGFB1, AR, and WIPI2 were predominantly expressed in cancer epithelial, CAF, and myeloid cells. The dot plot (Figure 15A) summarizes both the proportion of expressing cells (dot size) and the expression intensity (color scale).

Normal epithelial cells and CAFs showed the highest proportion and intensity of CIRBP expression, whereas endothelial cells exhibited strong FABP4 expression. Cancer epithelial cells displayed elevated WIPI2 and AR levels, while endothelial cells were the primary source of DECR1. DNAJB6 was most highly expressed in normal epithelial cells. In contrast, genes such as SLC25A28, TRIB2, SOX2, SIAH2, AKR1C2, and AKR1C3 appeared at low levels across most populations, although violin plots (Figure S6) revealed enrichment of AKR1C2, AKR1C3, and AR within cancer epithelial clusters, and FABP4 within endothelial cells.

UMAP visualization (Figure 15B) demonstrated clear spatial clustering of cell types, with cells sharing similar ferroptosis-related transcriptional profiles grouping together, while transcriptionally distinct populations were positioned farther apart.

**Figure 16:**
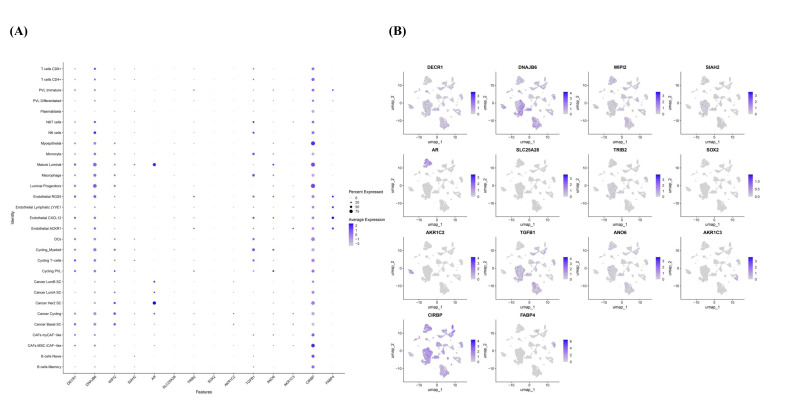

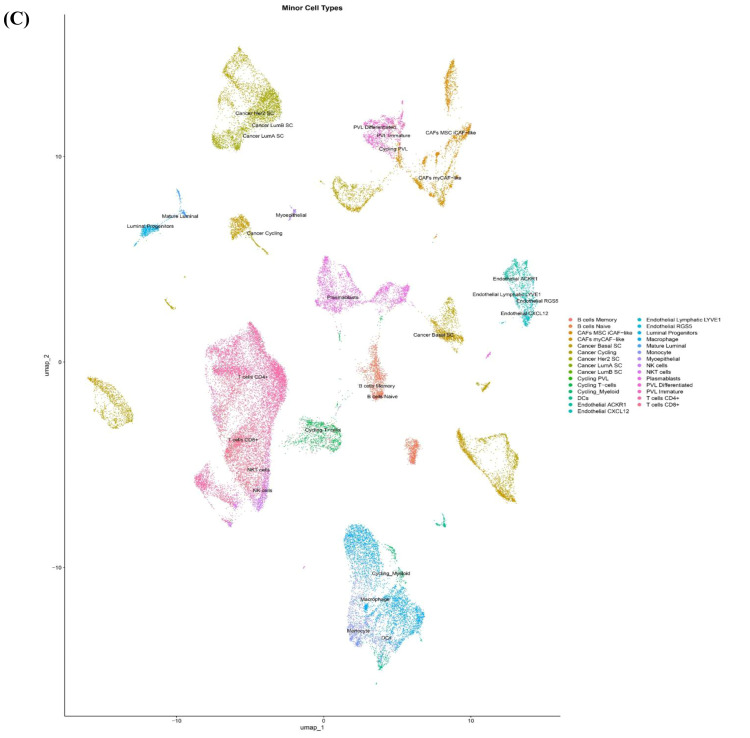
Expression of ferroptosis-related markers across minor cell subpopulations in TNBC (scRNA-seq, GSE176078). (**A**) Dot plot showing expression patterns across refined cellular subsets. (**B**) UMAP and (**C**) Seurat UMAP projections illustrating spatial clustering and distribution of ferroptosis-related transcripts. DECR1 and DNAJB6 are enriched in luminal, CAFs, endothelial, and cycling cell populations; AR is expressed in mature luminal and HER2-like cancer cells; TGFB1 is abundant in myeloid and macrophage subsets; CRIPBP shows broad expression across epithelial, stromal, and immune cells.

Minor cell type analysis provided additional resolution, revealing that ferroptosis-related genes display distinct expression patterns across refined cellular subpopulations (Figure 16A–C and Figure S7). DECR1 was prominently expressed in mature luminal cells, endothelial RGS5^+^ cells, CAFs, luminal progenitors, cycling T-cells, cancer basal and cancer cycling cells. DNAJB6 showed high abundance across mature luminal cells, CAFs, macrophages, luminal progenitors, cycling myeloid, endothelial RGS5, cycling T-cells, cycling PVL, cancer cycling, cancer basal SC. AR expression was enriched primarily in mature luminal cells and HER2-like cancer stem cells, whereas TGFB1 was predominantly expressed in myeloid-derived populations, including cancer-associated macrophages and monocytes. CIRBP showed broad expression and was detectable in nearly all analyzed subpopulations (Figure 16A–C and Figure S7).

Differences were also observed within cancer epithelial lineages and compared with normal mature luminal and luminal progenitor cells, cancer luminal A, luminal B, and HER2-enriched epithelial clusters displayed reduced levels of DECR1 and DNAJB6, suggesting subtype-specific differences in the expression of genes associated with ferroptosis-related metabolic and stress-response pathways (Figure 16A–C and Figure S7).

It is important to note that the single-cell RNA-seq analysis in this study is descriptive. No formal statistical testing of differential expression across cell types was conducted due to the limited number of TNBC samples in the scRNA-seq dataset (*n* = 10). Therefore, the observed cell-type-specific expression patterns of ferroptosis-related genes should be interpreted as exploratory and hypothesis-generating.

## 4. Discussion

Triple-negative breast cancer (TNBC) is the most aggressive breast cancer subtype, characterized by high-grade, early relapse, and poor survival. In the METABRIC cohort (that we used in our study), Basal tumors, the molecular equivalent of TNBC, showed the youngest patient age, the shortest overall survival, and the highest frequency of grade 3 and node-positive disease, supporting their selection for ferroptosis-focused transcriptional stratification analyses. Restricting the model to Basal tumors minimized confounding from hormonal and HER2 signaling and enabled identification of ferroptosis-associated survival genes with biological relevance to TNBC. The resulting gene signature was evaluated in TCGA and GEO cohorts. While METABRIC provided a sufficiently powered Basal-like discovery cohort, external evaluation using TCGA and GEO datasets was constrained by limited representation of PAM50 Basal-like tumors in TCGA and the absence of PAM50-based subtype annotation in GEO. Consequently, external evaluation was performed at the cohort level to assess reproducibility of ferroptosis-associated transcriptional patterns rather than to establish definitive subtype-specific clinical validation. The observation of statistically significant survival differences in the TCGA cohort, despite limited subtype-specific power, supports the reproducibility and biological consistency of the ferroptosis-associated transcriptional patterns identified in METABRIC. Importantly, the modest AUC values indicate that this approach is not intended to serve as an independent clinical predictor, but rather as a biologically informative framework that captures ferroptosis-associated transcriptional states linked to survival-associated risk stratification in Basal-like breast cancer.

We performed an integrative analysis of 509 ferroptosis-related genes from the literature [24] and online databases (FerrDb V2) [23]. This analysis revealed distinct expression patterns across breast cancer subtypes. Within the METABRIC cohort, Basal-like tumors demonstrated marked upregulation of LCN2, NDRG1, CA9, CBS, CDKN2A, EZH2, KIF20A, and CDCA3, genes associated with iron metabolism, cell cycle progression, hypoxia adaptation, and proliferative signaling and metastasis [25,26,27,28,29,30,31,32]. Conversely, AR, SLC40A1, CIRBP, MUC1, FABP4, ELOVL5, and NQO1 were strongly downregulated, reflecting loss of hormonal signaling, iron export, lipid storage, and antioxidant capacity typical of TNBC. This coordinated shift promotes metabolic rewiring and redox imbalance, contributing to ferroptosis susceptibility in Basal-like tumors [33,34,35,36,37,38]. When comparing Luminal A versus Basal BC, Basal tumors display slight upregulation of genes primarily associated with iron metabolism (TF and LCN2), lipid storage (PLIN2 and LPIN1), and stress responses (MT1G, NDRG1, and IDO1), as well as growth factor signaling (EGFR), indicating enhanced ferroptotic susceptibility or regulatory adaptation in the Basal tumors. Similarly, when comparing Luminal B with Basal tumors, Basal tumors display slight upregulation of genes associated with oxidative stress regulation (MT1G, NDRG1), hypoxia signaling (CA9, NDRG1), iron and sulfur metabolism (TF, CBS, LCN2), lipid homeostasis (LPIN1, EGFR, NT5DC2), and cell cycle control (CDKN2A), collectively reflecting redox balance, metabolic adaptation, and proliferative signaling. This suggests that the Basal subtype exhibits a more pronounced ferroptosis-related gene signature than either Luminal A or Luminal B tumors. Basal-like tumors in comparison with HER-2 enriched tumors exhibit higher expression of genes associated with oxidative stress and metabolic adaptation, such as LCN2, CDKN2A, FZD7, and TF, reflecting a ferroptosis-primed phenotype characterized by redox imbalance and altered iron metabolism. Conversely, increased expression of SLC40A1, NQO1, AR, and MUC1 is observed in HER2-enriched tumors. These genes are involved in iron export, NAD(P)H-dependent antioxidant defense, and epithelial differentiation.

These expression patterns suggest that Basal-like tumors are more susceptible to ferroptosis induction, whereas HER2-enriched tumors retain partial antioxidant and iron handling capacity, potentially contributing to their differential therapeutic response.

Sixteen ferroptosis-related genes were associated with overall survival in Basal-like tumors. Protective genes (DECR1, WIPI2, AR, SLC25A28, TRIB2, AKR1C2, TGFB1, AKR1C3) participate in PUFA oxidation [39], autophagy [40], iron homeostasis [41], and redox regulation [42,43]. In contrast, high-risk genes (DNAJB6, SIAH2, STAT3, CGAS, EZH2, SOX2, H19, ANO6) are linked to tumor progression, inflammation, and ferroptosis resistance [44,45]. These candidates were combined into a 16-gene prognostic signature that provided supportive stratification of patients into high- and low-risk groups.

The Ferroptosis Index further highlighted a Basal-dominant ferroptosis-associated transcriptional state, consistent with the oxidative and metabolic stress phenotypes of these tumors, which may influence both disease aggressiveness and response to redox-targeted therapies. Importantly, the FI should not be interpreted as evidence of ongoing ferroptotic cell death. Instead, the elevated FI observed in Basal-like tumors reflects a ferroptosis-poised transcriptional and metabolic stress state, indicating increased susceptibility to ferroptosis under appropriate conditions. Functional enrichment analyses demonstrated that ferroptosis-related genes in Basal tumors are predominantly associated with cell cycle progression, chromosomal organization, and mitotic control, supporting a proliferative and checkpoint-deregulated phenotype. Luminal and HER2-enriched tumors were enriched for differentiation and cytoskeletal pathways, suggesting metabolic adaptations that blunt ferroptotic stress.

AR emerged as the only recurrent overlap across ferroptosis-related genes associated with survival and differentially expressed genes in multiple subtype comparisons, while EZH2 additionally overlapped in Basal vs. Normal-like tumors, implicating both genes as context-dependent modulators of ferroptosis. In METABRIC cohort, ACSL4 and EZH2 were consistently upregulated in Basal tumors, supporting enhanced ferroptosis-associated transcriptional potential, epigenetic activation, and pro-invasive molecular profile [46,47,48]. In contrast, AR, GPX4, and CIRBP were reduced, indicating impaired antioxidant defense and enhanced lipid peroxidation [49,50,51,52]. CPTAC proteomics confirmed these trends at the protein level when comparing protein expression between Basal and non-Basal tumors. However, when protein and mRNA expression were evaluated across individual molecular subtypes, notable discrepancies emerged, indicating subtype-specific divergence between transcriptional and proteomic regulation. ACSL4 and EZH2 were highest in Basal tumors, while AR and CIRBP were lowest in Basal tumors and highest in Luminal tumors, reflecting hormone receptor loss and reduced stress tolerance. ANO6 is highest in Luminal A and Normal-like subtypes, while Basal tumors showed slightly lower levels, indicating subtype-specific membrane remodeling [53]. However, GPX4 was lowest in Basal and highest in Luminal tumors, supporting diminished ferroptosis resistance in TNBC, while WIPI2 was moderately elevated in Basal and HER2-enriched tumors, suggesting increased autophagy-ferroptosis coupling [54]. SLC7A11 was generally low except in Luminal B, where it was markedly upregulated, indicating enhanced antioxidant capacity [55]. The proteomic analysis across PAM50 subtypes highlights Basal tumors as ferroptosis-prone, epigenetically driven, and invasive, marked by high ACSL4 and EZH2, and low ANO6, AR, GPX4, and CIRBP.

Survival analysis further supported these findings, showing that EZH2 and AR overexpression, as well as low CIRBP levels, were significantly associated with poor overall survival, highlighting the interplay between ferroptosis regulation, epigenetic remodeling, hormone signaling, and clinical outcome.

Previous studies have used single-cell analyses to explore cellular heterogeneity associated with ferroptosis sensitivity in various tumor types [56,57,58]. In our in silico study, single-cell analyses mapped ferroptosis-related transcripts across epithelial, CAF, endothelial, and immune populations, indicating that ferroptosis-associated transcriptional signals are distributed across both cancer cells and components of the tumor microenvironment in TNBC. While our single-cell analyses suggest that ferroptosis-related transcripts are present across stromal and immune compartments, these observations do not imply functional regulation of ferroptosis by these cell types. Rather, they highlight potential cellular contributors to ferroptosis-associated signaling within the tumor microenvironment, which warrant further functional investigation.

These findings suggest that two genes, AR and EZH2, exemplify the biological significance of ferroptosis-related factors in breast cancer. The androgen receptor, although typically associated with hormone receptor-positive disease, is variably expressed in TNBC and may exert context-dependent effects on tumor progression and therapy response [59]. EZH2, a histone methyltransferase and a key component of the polycomb repressive complex 2, has been implicated in chromatin remodeling, stemness, and aggressive tumor behavior, and its overexpression correlates with poor prognosis in TNBC [48]. The androgen receptor can promote ferroptosis resistance by directly inducing GPX4, a key lipid repair enzyme, explaining reduced sensitivity in LAR (Luminal Androgen Receptor) tumors [60]. Although AR can promote PUFA accumulation by downregulating DECR1, its dominant effect is to upregulate GPX4, making LAR tumors resistant to ferroptosis [60]. This dual role explains the limited clinical efficacy of AR inhibitors; AR blockade lowers GPX4 while also reducing PUFA-driven lipid peroxidation [60]. Consequently, direct GPX4 inhibition represents a more effective therapeutic approach in LAR TNBC.

Conversely, EZH2 acts as an epigenetic suppressor of ferroptosis by repressing TFR2 and lowering intracellular iron [61]. At the same time, EZH2 inhibition triggers compensatory upregulation of GPX4/SLC7A11 and GSH synthesis, which temporarily protects cells from lipid peroxidation-driven ferroptosis [62], creating a GPX4-dependent vulnerability. In the same model, EZH2 inhibitors also increase MUFA and PUFA abundance by upregulating SCD1 and ELOVL2, thereby enriching membranes with peroxidation-prone fatty acids while simultaneously activating GPX4-dependent repair, which constrains ferroptosis [62]. Together, these findings position EZH2 at the intersection of chromatin control, lipid metabolism, and ferroptosis resistance, suggesting that co-targeting EZH2 and GPX4 may be therapeutically beneficial in TNBC.

While AR downregulation and EZH2 upregulation are well-established hallmarks of TNBC biology, the novelty of our findings lies in positioning these factors within a ferroptosis-associated transcriptional landscape in Basal-like tumors. Our integrative analyses suggest that AR and EZH2 define distinct ferroptosis-related transcriptional states, linking hormone signaling and epigenetic regulation to redox balance and ferroptosis susceptibility rather than serving solely as subtype identifiers. Importantly, proposed mechanistic links are hypothesis-generating and based on integration of transcriptomic patterns with the prior literature rather than direct functional validation within this study.

Cysteine availability further influences ferroptotic vulnerability in TNBC. Cystine deprivation depletes glutathione (GSH), increases ROS, and triggers necroptosis and ferroptosis [63], while TNBC cells intrinsically contain lower GSH and GSS levels than non-TNBC tumors, making them more sensitive to oxidative stress [12,64]. LAR tumors show elevated GSH-cycle metabolites, indicating a strong dependence on GSH metabolism as a key ferroptosis-suppressive mechanism

Overall, TNBC cells harbor a distinct ferroptosis-associated program characterized by high ferroptosis-associated transcriptional potential, proliferative drive, and immune remodeling. These features may underlie their clinical aggressiveness and highlight ferroptosis-related genes as candidate biomarkers and therapeutic targets.

This study has several limitations. First, the analyses relied on retrospective public datasets and the lack of functional validation for key regulators such as AR, EZH2, CIRBP. Repetition of these findings in independent patient cohorts and prospective datasets will be required to confirm their robustness and universality.

Second, model coefficients derived from the METABRIC cohort were applied unchanged to the TCGA and GEO datasets, without retraining or parameter optimization. Accordingly, external evaluation should be interpreted as supportive rather than confirmatory, and differences in cohort size and composition may have influenced evaluation performance.

Survival analyses were primarily based on univariate Cox regression, as the primary objective was to identify ferroptosis-associated transcriptional programs linked to survival-associated risk stratification rather than to establish an independent clinical prognostic model. Given heterogeneity in treatment annotation and cohort composition across publicly available datasets, robust multivariable modeling and benchmarking against established clinical variables or existing TNBC gene signatures will require harmonized prospective datasets and should be addressed in future studies.

Finally, the single-cell RNA-seq analysis was descriptive and hypothesis-generating, without formal statistical testing or functional validation across cell types.

This in silico study provides valuable insights into the complex signaling pathways underlying TNBC progression and the aggressive, invasive, and metastatic potential associated with alterations in ferroptosis signaling. These analyses revealed novel aspects of the heterogeneous BC pathology and may be used to sub-stratify TNBC patients into groups of patients who are candidates for additional targeted therapy, thereby increasing effectiveness and complementing standard treatment. This research, if confirmed in vitro and in animal models, may provide a solid basis for designing a clinical study focused on the sub-classification of ferroptosis-sensitive tumors and, in the next phase, testing drugs that enhance the activity of crucial ferroptosis inducers.

## 5. Conclusions

This study identifies ferroptosis-related molecular signatures with biological stratification potential in triple-negative breast cancer, particularly within the Basal-like subtype. Through integrated transcriptomic, proteomic, and single-cell analyses, we demonstrate that Basal-like tumors exhibit a distinct ferroptosis-associated transcriptional state characterized by metabolic and redox stress rather than evidence of ongoing ferroptotic cell death. Our findings highlight AR and EZH2 as context-dependent components embedded within ferroptosis-related transcriptional networks, linking hormone signaling and epigenetic regulation to ferroptosis susceptibility. Importantly, these associations are hypothesis-generating and based on integrative in silico analyses rather than direct functional validation.

Collectively, this work provides a biologically grounded framework for understanding ferroptosis-associated heterogeneity in aggressive breast cancer and offers a rationale for future mechanistic and translational studies aimed at exploiting ferroptosis-related vulnerabilities in TNBC.

## Data Availability

All datasets used in this study (METABRIC, TCGA, GEO, CPTAC, and GSE176078) are publicly available from cBioPortal, GEPIA, UCSC Xena, CPTAC Data Portal, and GEO (NCBI). METABRIC data sources were accessed on 23 January 2025; TCGA data sources were accessed on 27 January 2025; GEO data sources were accessed on 28 January 2025; CPTAC23 data sources were accessed in September 2025; GSE176078 data sources were accessed on 4 February 2025.

## Supplementary Materials

The following supporting information can be downloaded at https://www.mdpi.com/article/10.3390/diagnostics16030379/s1, Figure S1: Expression heatmap of ferroptosis-related genes in basal-like, Luminal A, Luminal B and HER-2 enriched tumors; Figure S2: Construction and evaluation of the ferroptosis-based prognostic model for TNBC; Figure S3: Construction and evaluation of the ferroptosis-based prognostic model for TNBC; Figure S4: Principal component analysis dot plots for selected prognostic genes; Figure S5A: Functional enrichment analysis of consensus ferroptosis-related genes; Figure S5B: Functional enrichment analysis of consensus ferroptosis-related genes; Figure S5C: Functional enrichment analysis of consensus ferroptosis-related genes; Figure S5D: Functional enrichment analysis of consensus ferroptosis-related genes; Figure S6: Expression of ferroptosis-related markers across major cell types in TNBC (scRNA-seq, GSE176078); Figure S7: Expression of ferroptosis-related markers across minor cell subpopulations in TNBC (scRNA-seq, GSE176078); Table S1: Baseline clinical characteristics of BC patients from METABRIC datasets in the study; Table S2: Basal_vs_Normal_significant_fer_deg_genes-Supplementary; Table S3: Basal_vs_LumA_significant_fer_deg_genes-Supplementary; Table S4: Basal_vs_LumB_significant_fer_deg_genes; Table S5: Basal_vs_Her2_significant_fer_deg_genes; Table S6: Basal_selected_genes_lasso_deg_prognostic.

## Abbreviations

The following abbreviations are used in this manuscript:

ACSL4	Acyl-CoA synthetase long-chain family member 4
AR	Androgen receptor
Basal-like	Basal subtype of breast cancer
BC	Breast cancer
CAF(s)	Cancer-associated fibroblast(s)
CPTAC	Clinical Proteomic Tumor Analysis Consortium
DEG(s)	Differentially expressed gene(s)
ECM	Extracellular matrix
ER	Estrogen receptor
EZH2	Enhancer of zeste homolog 2
FI	Ferroptosis Index
FRG(s)	Ferroptosis-related gene(s)
GEO	Gene Expression Omnibus
GO	Gene Ontology
GPX4	Glutathione peroxidase 4
GSEA	Gene Set Enrichment Analysis
GSH	Glutathione
HER2-enriched	HER2-positive molecular subtype
HR	Hazard ratio
KEGG	Kyoto Encyclopedia of Genes and Genomes
LAR	Luminal Androgen Receptor subtype of triple-negative breast cancer
LumA	Luminal A subtype
LumB	Luminal B subtype
METABRIC	Molecular Taxonomy of Breast Cancer International Consortium
NC	Not classified
PAM50	Prediction Analysis of Microarray 50-gene classifier
PCA	Principal component analysis
PR	Progesterone receptor
scRNA-seq	Single-cell RNA sequencing
SLC7A11	Solute carrier family 7 member 11 (xCT)
TCGA	The Cancer Genome Atlas
TME	Tumor microenvironment
TNBC	Triple-negative breast cancer
t-SNE	t-distributed stochastic neighbor embedding
UMAP	Uniform Manifold Approximation and Projection
