# Supplementary material for: The Landscape of Ferroptosis-Related Gene Signatures as Molecular Stratification in Triple-Negative Breast Cancer
Source: Diagnostics (Basel). 2026 Jan 23;16(3):379. doi: 10.3390/diagnostics16030379 (PMC12896675; doi:10.3390/diagnostics16030379)
Supplement: Supplementary file 1 [file diagnostics-16-00379-s001.zip › diagnostics-4062002-supplementary.pdf]

Ferroptosis-Related Gene Signatures as Potential Diagnostic and Prognostic Markers in Triple-Negative Breast Cancer

Marko Buta[1,2†], Nikola Jeftic[1†], Irina Besu[3], Jovan Raketic[1], Ivan Markovic[1,2], Ana Djuric[3], Nina Petrovic[3], Tatjana Srdic-Rajic[3]

[1]Clinic of Surgical Oncology, Institute for Oncology and Radiology of Serbia, Belgrade, Serbia
[2]School of Medicine, University of Belgrade, Belgrade, Serbia
[3] Department of Experimental Oncology, Institute for Oncology and Radiology of Serbia, Belgrade, Serbia

[*]Corresponding authors:
Nina Petrovic:nina.petrovic@ncrc.ac.rs
Tatjana Srdic-Rajic: tatjana.srdic@ncrc.ac.rs; tsrdic@gmail.com

[†]These authors contributed equally to this work.

CONTENT

Baseline clinical characteristics of BC patients from METABRIC datasets in the study-Table S1..............S2

Expression heatmap of ferroptosis-related genes in basal-like, Luminal A, Luminal B and HER-2 enriched tumors-Figure S1.............S3

Construction and evaluation of the ferroptosis-based prognostic model for TNBC-Figure S2....................S4

Construction and evaluation of the ferroptosis-based prognostic model for TNBC-Figure S3....................S5

Principal component analysis dot plots for selected prognostic genes-Figure S4...S6

Functional enrichment analysis of consensus ferroptosis-related genes-Figure S5A.....................................S7

Functional enrichment analysis of consensus ferroptosis-related genes-Figure S5B.....................................S7

Functional enrichment analysis of consensus ferroptosis-related genes-Figure S5C......................................S8

Functional enrichment analysis of consensus ferroptosis-related genes-Figure S5D......................................S8

Expression of ferroptosis-related markers across major cell types in TNBC (scRNA-seq, GSE176078)-Figure S6..............S9

Expression of ferroptosis-related markers across minor cell subpopulations in TNBC (scRNA-seq, GSE176078)-Figure S7..............S10

Basal_vs_Normal_significant_fer_deg_genes-Supplementary-Table S2.

Basal_vs_LumA_significant_fer_deg_genes-Supplementary Table S3.

Basal_vs_LumB_significant_fer_deg_genes-Supplementary Table S4

Basal_vs_Her2_significant_fer_deg_genes-Supplementary Table S5.

Basal_selected_genes_lasso_deg_prognostic-Supplementary Table S6.

Table S1. **Baseline clinical characteristics of BC patients from METABRIC datasets in the study.**

Characteristic	claudin-low	LumA	LumB	Normal	Her2	Basal	NC
ER	Negative: 138; Positive: 80	Positive: 696; Negative: 4	Positive: 475	Positive: 125; Negative: 23	Negative: 129; Positive: 95	Negative: 180; Positive: 29	Positive: 6
N	218	700	475	148	224	209	6
Radio Therapy	YES: 145; NO: 73	YES: 378; NO: 322	YES: 299; NO: 176	YES: 74; NO: 74	YES: 134; NO: 90	YES: 141; NO: 68	NO: 4; YES: 2
Hormone Therapy	YES: 110; NO: 108	YES: 477; NO: 223	YES: 379; NO: 96	YES: 89; NO: 59	NO: 127; YES: 97	NO: 149; YES: 60	YES: 4; NO: 2
PR	Negative: 178; Positive: 40	Positive: 539; Negative: 161	Positive: 299; Negative: 176	Positive: 90; Negative: 58	Negative: 174; Positive: 50	Negative: 191; Positive: 18	Positive: 4; Negative: 2
Tumor size (median, range)	20.0 (2.0-100.0)	20.0 (1.0-150.0)	25.0 (5.0-180.0)	23.0 (1.0-120.0)	25.0 (1.0-160.0)	25.0 (1.0-182.0)	17.5 (15.0-35.0)
Chemotherapy	NO: 145; YES: 73	NO: 644; YES: 56	NO: 428; YES: 47	NO: 113; YES: 35	NO: 136; YES: 88	YES: 113; NO: 96	NO: 6
Grade	3.0: 136; 2.0: 53; 1.0: 15	2.0: 378; 3.0: 172; 1.0: 118	3.0: 253; 2.0: 186; 1.0: 18	2.0: 79; 3.0: 48; 1.0: 12	3.0: 155; 2.0: 54; 1.0: 4	3.0: 187; 2.0: 17; 1.0: 2	2.0: 4; 3.0: 1
Tumor stage	2.0: 86; 1.0: 64; 3.0: 21; 0.0: 5	2.0: 286; 1.0: 222; 3.0: 26; 4.0: 2	2.0: 210; 1.0: 104; 3.0: 30; 4.0: 6; 0.0: 2	2.0: 58; 1.0: 42; 3.0: 5; 4.0: 2	2.0: 90; 1.0: 34; 3.0: 22; 0.0: 2	2.0: 92; 1.0: 35; 3.0: 14; 0.0: 3	2.0: 3
OS months (median)	116.2	130.1	104.1	121.1	97.1	85.5	144.1
HER2	Negative: 201; Positive: 17	Negative: 679; Positive: 21	Negative: 430; Positive: 45	Negative: 134; Positive: 14	Positive: 128; Negative: 96	Negative: 187; Positive: 22	Negative: 6
Node positive (%)	94/218 (43.1%)	287/700 (41.0%)	237/475 (49.9%)	56/148 (37.8%)	126/224 (56.2%)	107/209 (51.2%)	4/6 (66.7%)
Age (median, IQR)	58.7 (49.1-66.6)	63.4 (53.2-72.2)	66.5 (58.9-74.0)	57.1 (46.4-66.2)	59.1 (50.4-68.6)	54.2 (43.5-65.4)	72.1 (71.1-76.1)

Abbreviations (PAM50 molecular subtypes): Claudin-low; LumA-Luminal A; LumB-Luminal B; Normal-Normal-like; Her2HER2-enriched; Basal- Basal-like; NC-not classified

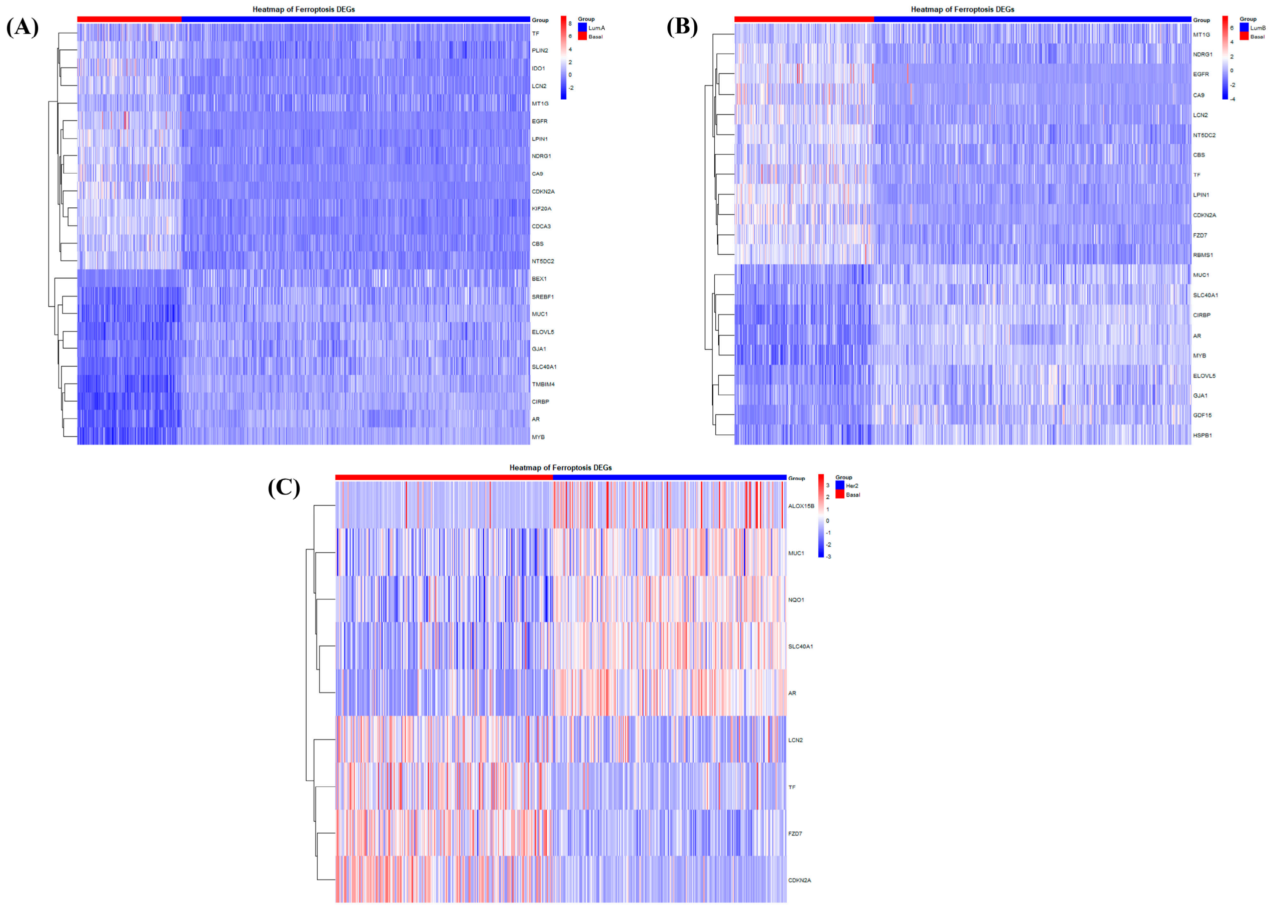

Figure S1. Expression heatmap of ferroptosis-related genes in Basal-like versus Luminal A, Luminal B and HER-2 enriched tumors. Heatmap illustrating the expression patterns of ferroptosis-related genes across Basal-like (red) and Luminal A (blue) samples (A), Basal-like (red) and Luminal B (blue) samples (B), Basal-like (red) and HER-2 enriched (blue) samples (C), showing clear group-level segregation and a distinct ferroptosis-associated signature enriched in Basal-like tumors.

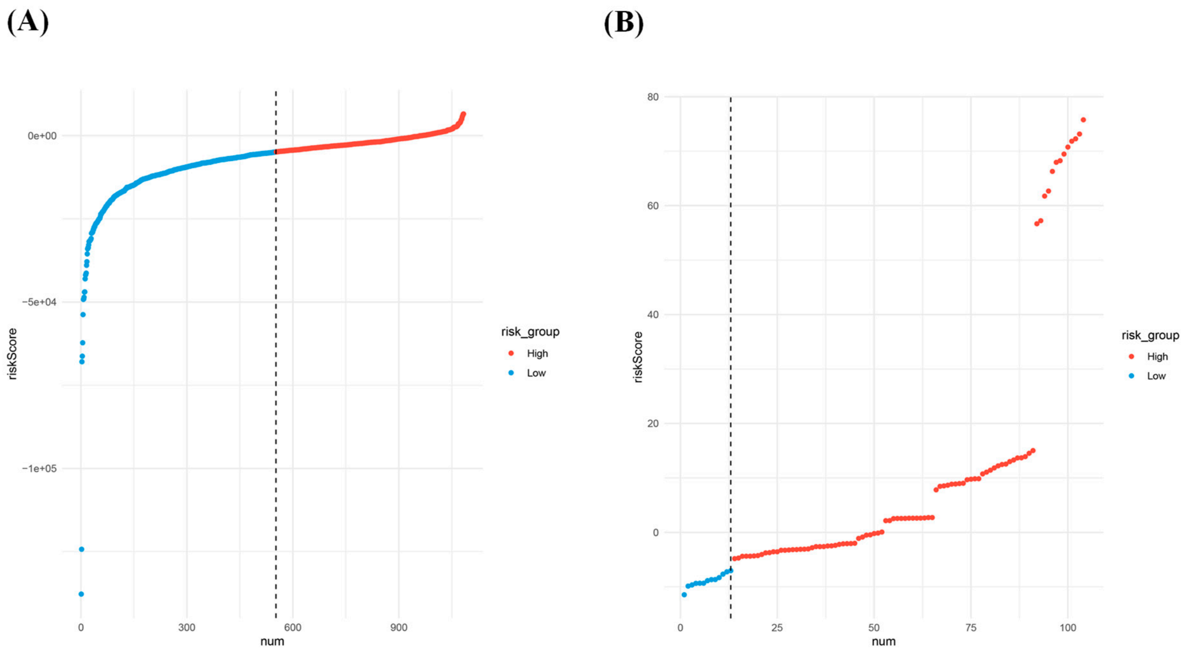

Figure S2. Construction and evaluation of the ferroptosis-associated transcriptional stratification framework for Basal-like tumors. Risk score distribution. Breast cancer patients were divided into low and high-risk score groups according to cut-off values in (A) TCGA evaluation cohort and (B) GEO evaluation cohort.

(A)

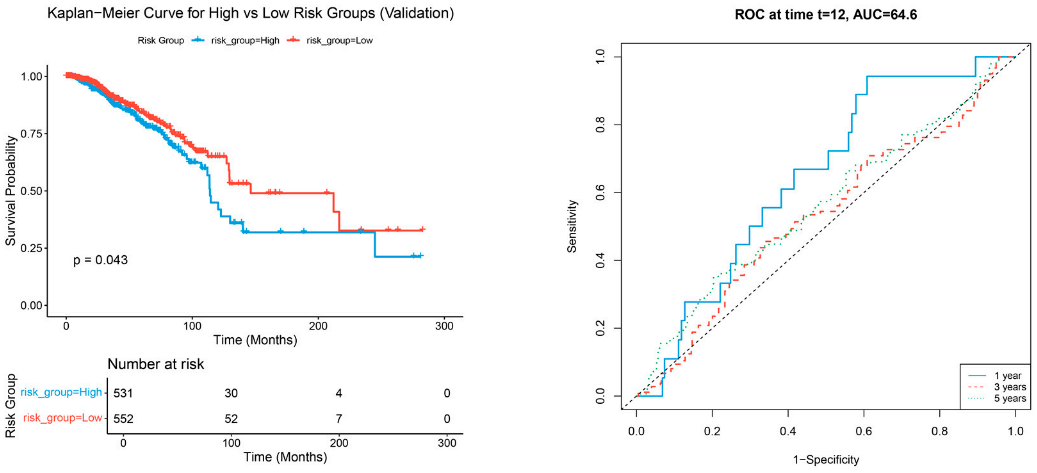

(B)

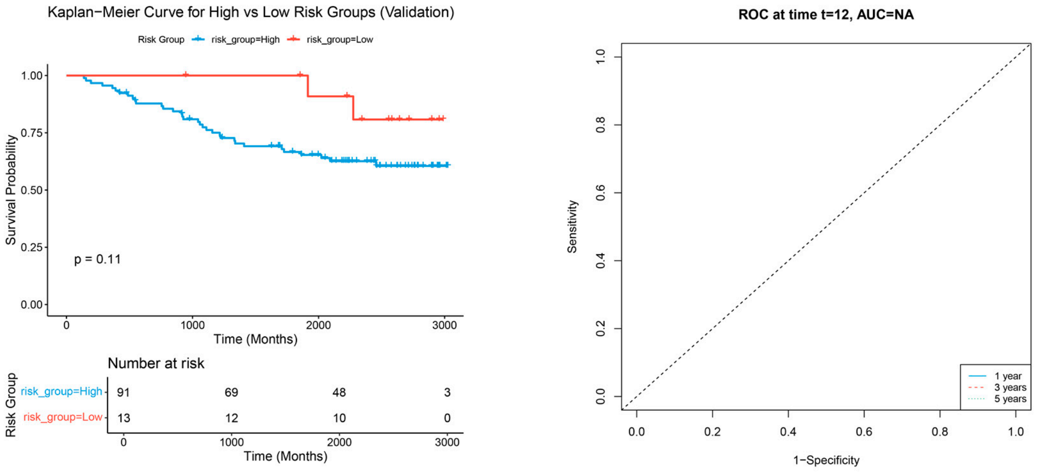

Figure S3. Construction and evaluation of the ferroptosis-based prognostic model for TNBC. Breast cancer patients were divided into low- and high-risk groups based on the METABRIC cohort cut-off values. Kaplan Meier analysis-left and Time-dependent ROC curves-right for (A) TCGA evaluation cohort, and (B) GEO evaluation cohort.

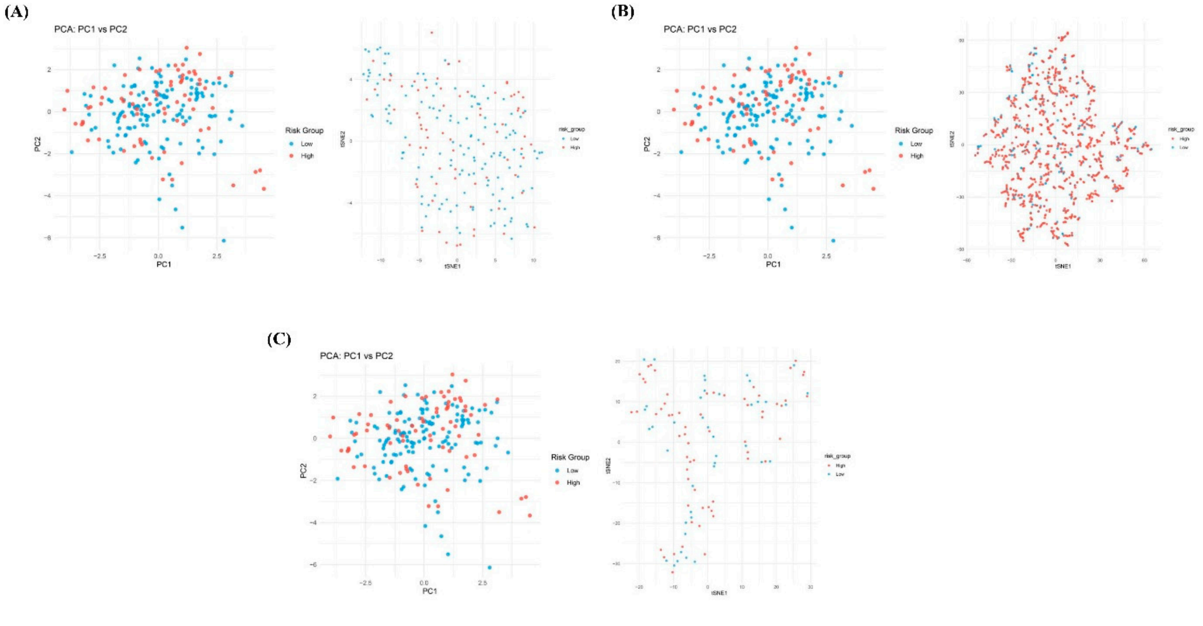

Figure S4. Principal component analysis dot plots for selected prognostic genes. Blue dots belong to the low-risk group, while red dots represent the high-risk group. A-C represent analysis and comparison of the first principal component vs second PC1 vs PC2 and t-SNE dot plots for selected prognostic genes (A) for METABRIC Basal cohort, TCGA evaluation cohort (B) and GEO evaluation cohort (C).

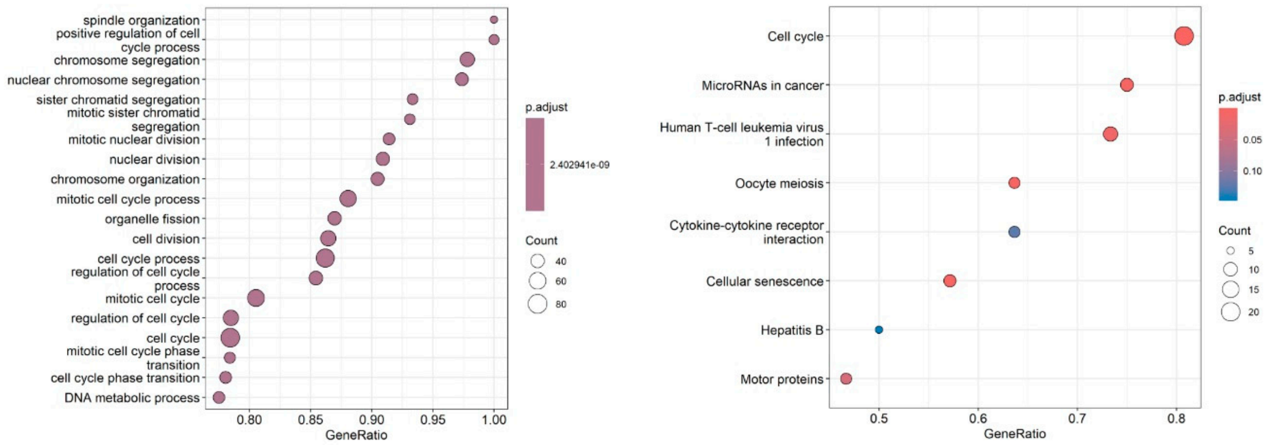

Figure S5A. Functional enrichment analysis of consensus ferroptosis-related genes for Basal versus Normal-like tumors. GO biological process identifies processes involving enrichment in the cell cycle process, chromosome segregation, and mitotic nuclear division (left), and KEGG pathways enrichment indicates in cell cycle, microRNAs in cancer, and cellular senescence, indicating links between ferroptosis, proliferation, chromosomal dynamics and oncogenic signaling in Basal-like tumors (right).

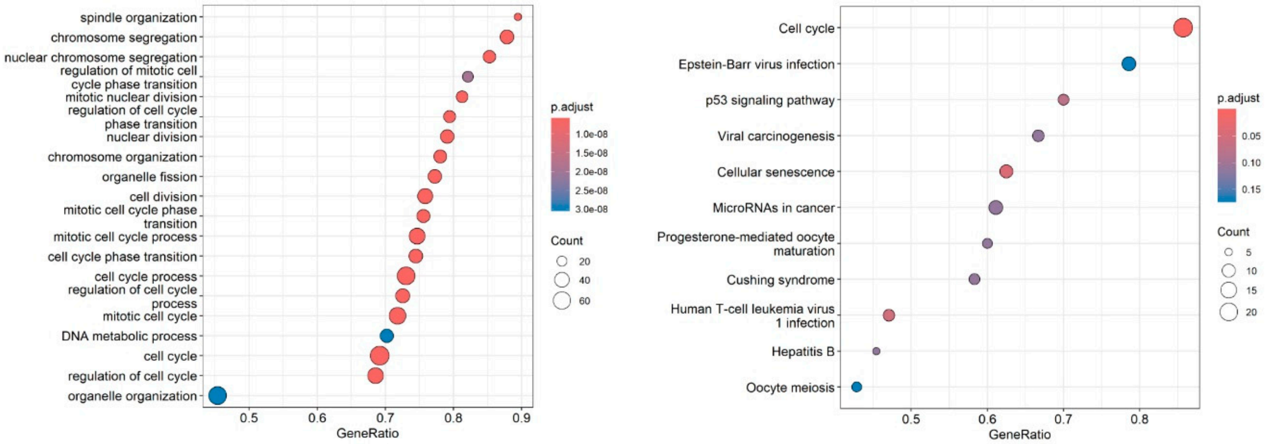

Figure S5B. Functional enrichment analysis of consensus ferroptosis-related genes for Basal versus Luminal A tumors. GO biological process identifies processes involving enrichment in cell cycle process, chromosome segregation, mitotic division (left) and KEGG pathways enrichment indicates in cell cycle, cellular senescence, p53 signaling pathway indicateed link between ferroptosis-related genes in Basal tumors with enhanced cell division, chromosomal organization, and DNA metabolism (right).

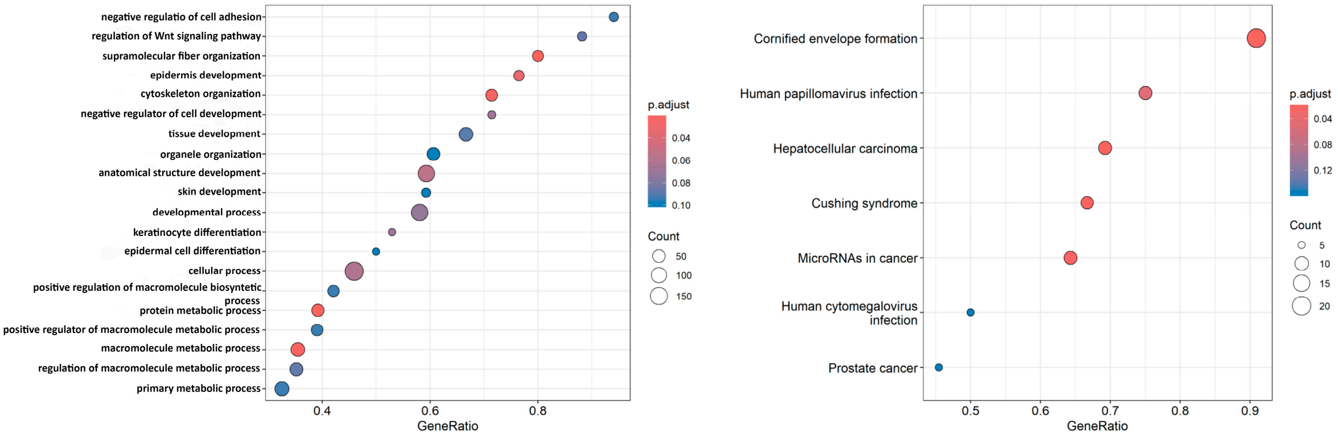

Figure S5C. Functional enrichment analysis of consensus ferroptosis-related genes for Basal versus Luminal B tumors. GO biological process identifies processes involving enrichment in supramolecular fiber organization, cytoskeleton organization, and epidermis development, suggesting roles in structural remodeling, differentiation, and metabolic re-programming (left). KEGG pathways enrichment indicates in Cushing syndrome, hepatocellular carcinoma, and microRNAs in cancer support the involvement of ferroptosis in stress response and oncogenic signaling (right).

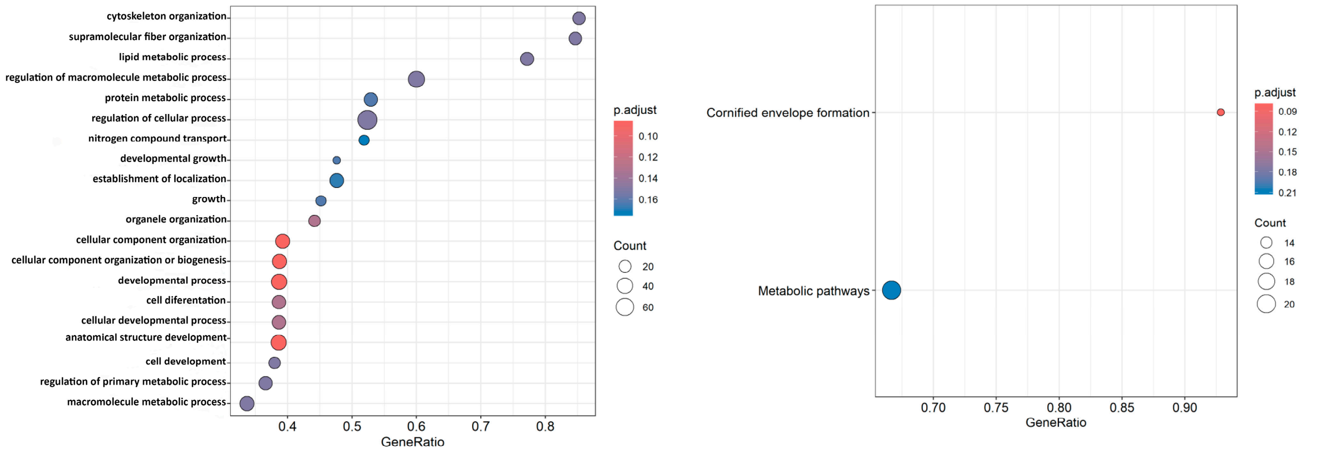

Figure S5D. Functional enrichment analysis of consensus ferroptosis-related genes for Basal versus HER-2 enriched tumors. GO biological process identifies processes involving enrichment in development and organelle organization(left). KEGG pathways enrichment indicates in cornified envelope formation and metabolic pathways (right) supporting the contribution of ferroptosis-related genes in Basal tumors to developmental and metabolic adaptation.

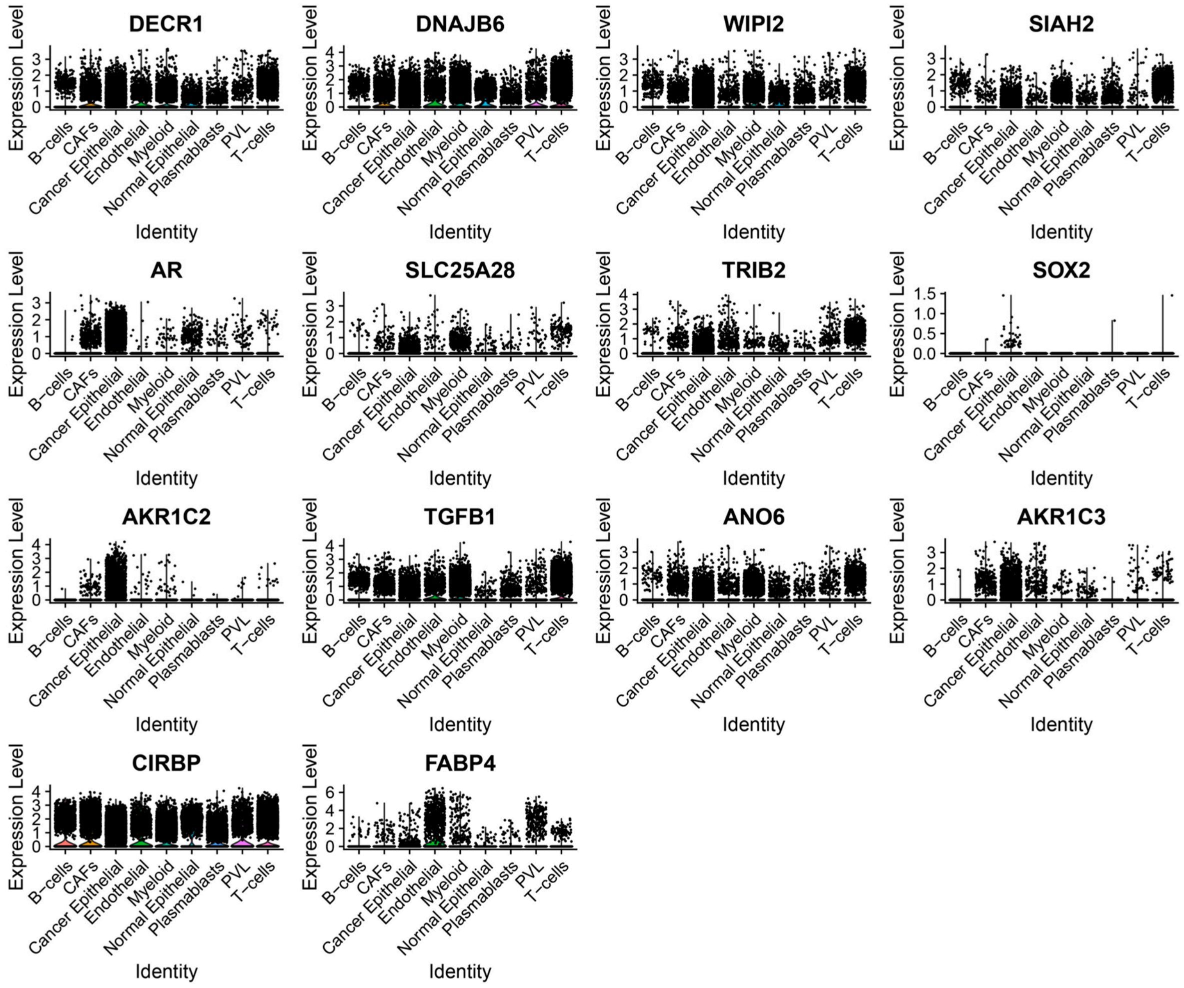

Figure S6. Expression of ferroptosis-related markers across major cell types in TNBC (scRNA-seq, GSE176078). Violin plots showed enrichment of AKR1C2, AKR1C3, and AR within cancer epithelial clusters, and FABP4 within endothelial cells.

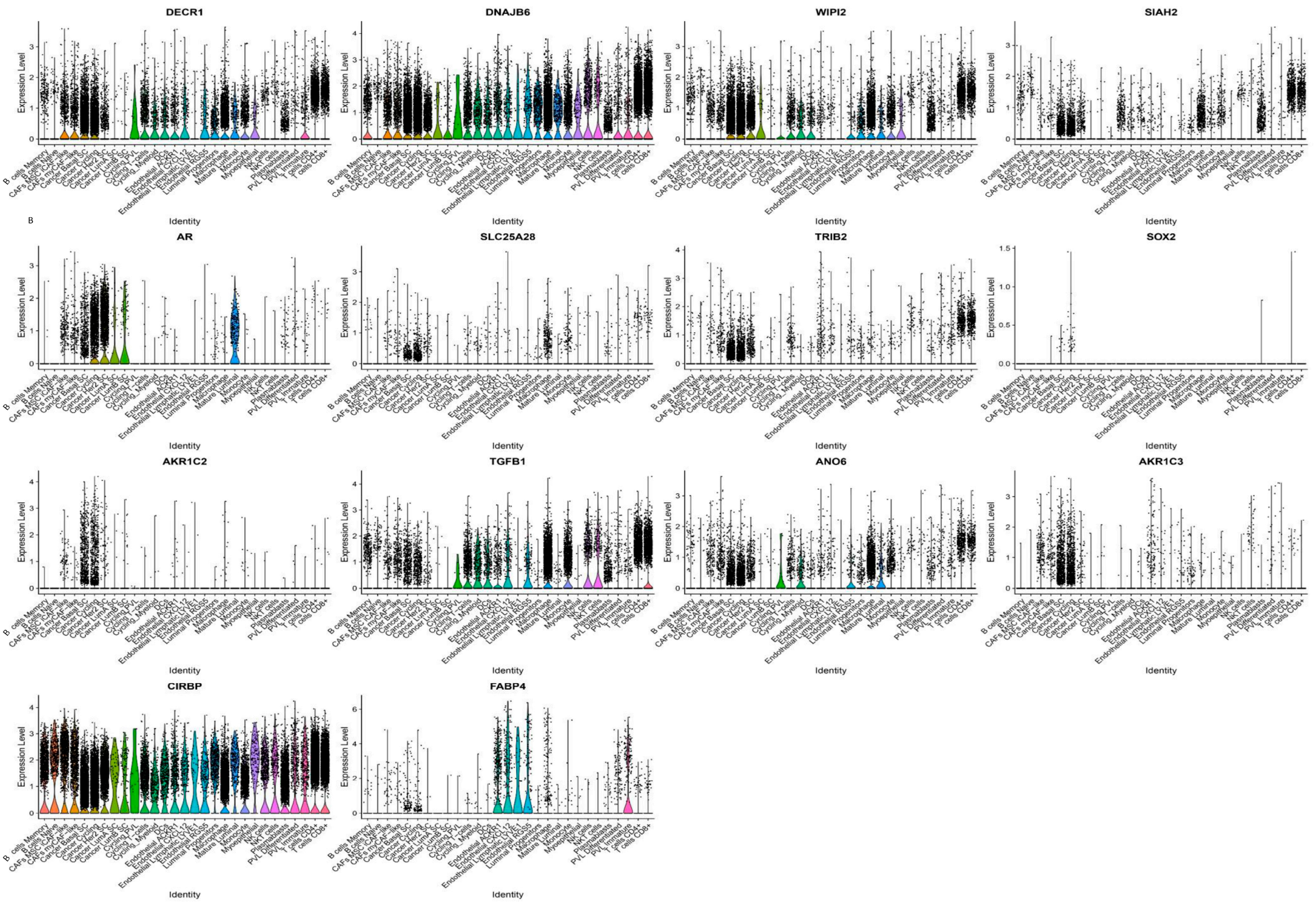

Figure S7. Expression of ferroptosis-related markers across minor cell subpopulations in TNBC (scRNA-seq, GSE176078). Violin plots showed that DECR1 and DNAJB6 are enriched in luminal, endothelial, CAFs, and cycling cell populations; AR is expressed in mature luminal and HER2-like cancer cells; TGFB1 is abundant in myeloid and macrophage subsets; CRIPBP shows broad expression across epithelial, stromal, and immune cells.
